# Bottleneck Problems: An Information and Estimation-Theoretic View †

**DOI:** 10.3390/e22111325

**Published:** 2020-11-20

**Authors:** Shahab Asoodeh, Flavio P. Calmon

**Affiliations:** School of Engineering and Applied Science, Harvard University, Cambridge, MA 02138, USA; flavio@seas.harvard.edu

**Keywords:** information bottleneck, privacy funnel, mutual information, data processing inequality

## Abstract

Information bottleneck (IB) and privacy funnel (PF) are two closely related optimization problems which have found applications in machine learning, design of privacy algorithms, capacity problems (e.g., Mrs. Gerber’s Lemma), and strong data processing inequalities, among others. In this work, we first investigate the functional properties of IB and PF through a unified theoretical framework. We then connect them to three information-theoretic coding problems, namely hypothesis testing against independence, noisy source coding, and dependence dilution. Leveraging these connections, we prove a new cardinality bound on the auxiliary variable in IB, making its computation more tractable for discrete random variables. In the second part, we introduce a general family of optimization problems, termed “bottleneck problems”, by replacing mutual information in IB and PF with other notions of mutual information, namely *f*-information and Arimoto’s mutual information. We then argue that, unlike IB and PF, these problems lead to easily interpretable guarantees in a variety of inference tasks with statistical constraints on accuracy and privacy. While the underlying optimization problems are non-convex, we develop a technique to evaluate bottleneck problems in closed form by equivalently expressing them in terms of lower convex or upper concave envelope of certain functions. By applying this technique to a binary case, we derive closed form expressions for several bottleneck problems.

## 1. Introduction

Optimization formulations that involve information-theoretic quantities (e.g., mutual information) have been instrumental in a variety of learning problems found in machine learning. A notable example is the information bottleneck (IB) method [1]. Suppose *Y* is a target variable and *X* is an observable correlated variable with joint distribution $P_{XY}$. The goal of IB is to learn a “compact” summary (aka bottleneck) *T* of *X* that is maximally “informative” for inferring *Y*. The bottleneck variable *T* is assumed to be generated from *X* by applying a random function *F* to *X*, i.e., T=F(X), in such a way that it is conditionally independent of *Y* given *X*, that we denote by

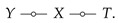
(1)
The IB quantifies this goal by measuring the “compactness” of *T* using the mutual information I(X;T) and, similarly, “informativeness” by I(Y;T). For a given level of compactness R≥0, IB extracts the bottleneck variable *T* that solves the constrained optimization problem

(2) IB(R)≔supI(Y;T)subjecttoI(X;T)≤R,

where the supremum is taken over all randomized functions T=F(X) satisfying *Y* 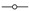 *X* 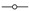 *T*.

The optimization problem that underlies the information bottleneck has been studied in the information theory literature as early as the 1970’s—see [2,3,4,5]—as a technique to prove impossibility results in information theory and also to study the common information between *X* and *Y*. Wyner and Ziv [2] explicitly determined the value of IB(R) for the special case of binary *X* and *Y*—a result widely known as Mrs. Gerber’s Lemma [2,6]. More than twenty years later, the information bottleneck function was studied by Tishby et al. [1] and re-formulated in a data analytic context. Here, the random variable *X* represents a high-dimensional observation with a corresponding low-dimensional feature *Y*. IB aims at specifying a compressed description of image which is maximally informative about feature *Y*. This framework led to several applications in clustering [7,8,9] and quantization [10,11].

A closely-related framework to IB is the privacy funnel (PF) problem [12,13,14]. In the PF framework, a bottleneck variable *T* is sought to maximally preserve “information” contained in *X* while revealing as little about *Y* as possible. This framework aims to capture the inherent trade-off between revealing *X* perfectly and leaking a sensitive attribute *Y*. For instance, suppose a user wishes to share an image *X* for some classification tasks. The image might carry information about attributes, say *Y*, that the user might consider as sensitive, even when such information is of limited use for the tasks, e.g., location, or emotion. The PF framework seeks to extract a representation of *X* from which the original image can be recovered with maximal accuracy while minimizing the privacy leakage with respect to *Y*. Using mutual information for both privacy leakage and informativeness, the privacy funnel can be formulated as

(3) PF(r)≔infI(Y;T)subjecttoI(X;T)≥r,

where the infumum is taken over all randomized function T=F(X) and *r* is the parameter specifying the level of informativeness. It is evident from the formulations (2) and (3) that IB and PF are closely related. In fact, we shall see later that they correspond to the upper and lower boundaries of a two-dimensional compact convex set. This duality has led to design of greedy algorithms [12,15] for estimating PF based on the agglomerative information bottleneck [9] algorithm. A similar formulation has recently been proposed in [16] as a tool to train a neural network for learning a private representation of data *X*; see [17,18] for other closely-related formulations. Solving IB and PF optimization problems analytically is challenging. However, recent machine learning applications, and deep learning algorithms in particular, have reignited the study of both IB and PF (see Related Work).

In this paper, we first give a cohesive overview of the existing results surrounding the IB and the PF formulations. We then provide a comprehensive analysis of IB and PF from an information-theoretic perspective, as well as a survey of several formulations connected to the IB and PF that have been introduced in the information theory and machine learning literature. Moreover, we overview connections with coding problems such as remote source-coding [19], testing against independence [20], and dependence dilution [21]. Leveraging these connections, we prove a new cardinality bound for the bottleneck variable in IB, leading to more tractable optimization problem for IB. We then consider a broad family of optimization problems by going beyond mutual information in formulations (2) and (3). We propose two candidates for this task: Arimoto’s mutual information [22] and *f*-information [23]. By replacing I(Y;T) and/or I(X;T) with either of these measures, we generate a family of optimization problems that we referred to as the bottleneck problems. These problems are shown to better capture the underlying trade-offs intended by IB and PF (see also the short version [24]). More specifically, our main contributions are listed next.

- Computing IB and PF are notoriously challenging when *X* takes values in a set with infinite cardinality (e.g., *X* is drawn from a continuous probability distribution). We consider three different scenarios to circumvent this difficulty. First, we assume that *X* is a Gaussian perturbation of *Y*, i.e., $X=Y+N^{G}$ where $N^{G}$ is a noise variable sampled from a Gaussian distribution independent of *Y*. Building upon the recent advances in entropy power inequality in [25], we derive a sharp upper bound for IB(R). As a special case, we consider jointly Gaussian (X,Y) for which the upper bound becomes tight. This then provides a significantly simpler proof for the fact that in this special case the optimal bottleneck variable *T* is also Gaussian than the original proof given in [26]. In the second scenario, we assume that *Y* is a Gaussian perturbation of *X*, i.e., $Y=X+N^{G}$. This corresponds to a practical setup where the feature *Y* might be perfectly obtained from a noisy observation of *X*. Relying on the recent results in strong data processing inequality [27], we obtain an upper bound on IB(R) which is tight for small values of *R*. In the last scenario, we compute second-order approximation of PF(r) under the assumption that *T* is obtained by Gaussian perturbation of *X*, i.e., $T=X+N^{G}$. Interestingly, the rate of increase of PF(r) for small values of *r* is shown to be dictated by an asymmetric measure of dependence introduced by Rényi [28].
- We extend the Witsenhausen and Wyner’s approach [3] for analytically computing IB and PF. This technique converts solving the optimization problems in IB and PF to determining the convex and concave envelopes of a certain function, respectively. We apply this technique to binary *X* and *Y* and derive a closed form expression for PF(r)– we call this result Mr. Gerber’s Lemma.
- Relying on the connection between IB and noisy source coding [19] (see [29,30]), we show that the optimal bottleneck variable *T* in optimization problem (2) takes values in a set T with cardinality |T|≤|X|. Compared to the best cardinality bound previously known (i.e., |T|≤|X|+1), this result leads to a reduction in the search space’s dimension of the optimization problem (2) from $R^{{\left|X\right|}^{2}}$ to $R^{\left|X|(|X|-1\right)}$. Moreover, we show that this does not hold for PF, indicating a fundamental difference in optimizations problems (2) and (3).
- Following [14,31], we study the deterministic IB and PF (denoted by dIB and dPF) in which *T* is assumed to be a deterministic function of *X*, i.e., T=f(X) for some function *f*. By connecting dIB and dPF with entropy-constrained scalar quantization problems in information theory [32], we obtain bounds on them explicitly in terms of |X|. Applying these bounds to IB, we obtain that $\frac{\mathrm{IB}(R)}{I(X;Y)}$ is bounded by one from above and by $\min\{\frac{R}{H(X)},\frac{e^{R}-1}{\left|X\right|}\}$ from below.
- By replacing I(Y;T) and/or I(X;T) in (2) and (3) with Arimoto’s mutual information or *f*-information, we generate a family of bottleneck problems. We then argue that these new functionals better describe the trade-offs that were intended to be captured by IB and PF. The main reason is three-fold: First, as illustrated in Section 2.3, mutual information in IB and PF are mainly justified when n≫1 independent samples $\left(X_{1},Y_{1}\right),\ldots ,\left(X_{n},Y_{n}\right)$ of $P_{XY}$ are considered. However, Arimoto’s mutual information allows for operational interpretation even in the single-shot regime (i.e., for n=1). Second, I(Y;T) in IB and PF is meant to be a proxy for the efficiency of reconstructing *Y* given observation *T*. However, this can be accurately formalized by probability of correctly guessing *Y* given *T* (i.e., Bayes risk) or minimum mean-square error (MMSE) in estimating *Y* given *T*. While I(Y;T) bounds these two measures, we show that they are precisely characterized by Arimoto’s mutual information and *f*-information, respectively. Finally, when $P_{XY}$ is unknown, mutual information is known to be notoriously difficult to estimate. Nevertheless, Arimoto’s mutual information and *f*-information are easier to estimate: While mutual information can be estimated with estimation error that scales as $O(\log n/\sqrt{n})$ [33], Diaz et al. [34] showed that this estimation error for Arimoto’s mutual information and *f*-information is $O(1/\sqrt{n})$.
  We also generalize our computation technique that enables us to analytically compute these bottleneck problems. Similar as before, this technique converts computing bottleneck problems to determining convex and concave envelopes of certain functions. Focusing on binary *X* and *Y*, we derive closed form expressions for some of the bottleneck problems.

### 1.1. Related Work

The IB formulation has been extensively applied in representation learning and clustering [7,8,35,36,37,38]. Clustering based on IB results in algorithms that cluster data points in terms of the similarity of $P_{Y|X}$. When data points lie in a metric space, usually geometric clustering is preferred where clustering is based upon the geometric (e.g., Euclidean) distance. Strouse and Schwab [31,39] proposed the deterministic IB (denoted by dIB) by enforcing that $P_{T|X}$ is a deterministic mapping: dIB(R) denotes the supremum of I(Y;f(X)) over all functions f:X→T satisfying H(f(X))≤R. This optimization problem is closely related to the problem of scalar quantization in information theory: designing a function f:X→[M]≔{1,…,M} with a pre-determined output alphabet with *f* optimizing some objective functions. This objective might be maximizing or minimizing H(f(X)) [40] or maximizing I(Y;f(X)) for a random variable *Y* correlated with *X* [32,41,42,43]. Since H(f(X))≤logM for f:X→[M], the latter problem provides lower bounds for dIB (and thus for IB). In particular, one can exploit [44] (Theorem 1) to obtain $I\left(X;Y\right)-\mathrm{dIB}\left(R\right)\leq O\left(e^{-2R/|Y|-1}\right)$ provided that $\min\{|X|,2^{R}\}>2|Y|$. This result establishes a linear gap between dIB and I(X;Y) irrespective of |X|.

The connection between quantization and dIB further allows us to obtain multiplicative bounds. For instance, if $Y∼\mathrm{Bernoulli}(\frac{1}{2})$ and $X=Y+N^{G}$, where $N^{G}∼N\left(0,1\right)$ is independent of *Y*, then it is well-known in information theory literature that $I\left(Y;f\left(X\right)\right)\geq \frac{2}{\pi }I\left(X;Y\right)$ for all non-constant f:X→{0,1} (see, e.g., [45] (Section 2.11)), thus $\mathrm{dIB}\left(R\right)\geq \frac{2}{\pi }I\left(X;Y\right)$ for R≤1. We further explore this connection to provide multiplicative bounds on dIB(R) in Section 2.5.

The study of IB has recently gained increasing traction in the context of deep learning. By taking *T* to be the activity of the hidden layer(s), Tishby and Zaslavsky [46] (see also [47]) argued that neural network classifiers trained with cross-entropy loss and stochastic gradient descent (SGD) inherently aims at solving the IB optimization problems. In fact, it is claimed that the graph of the function R↦IB(R) (the so-called the information plane) characterizes the learning dynamic of different layers in the network: shallow layers correspond to maximizing I(Y;T) while deep layers’ objective is minimizing I(X;T). While the generality of this claim was refuted empirically in [48] and theoretically in [49,50], it inspired significant follow-up studies. These include (i) modifying neural network training in order to solve the IB optimization problem [51,52,53,54,55]; (ii) creating connections between IB and generalization error [56], robustness [51], and detection of out-of-distribution data [57]; and (iii) using IB to understand specific characteristic of neural networks [55,58,59,60].

In both IB and PF, mutual information poses some limitations. For instance, it may become infinity in deterministic neural networks [48,49,50] and also may not lead to proper privacy guarantee [61]. As suggested in [55,62], one way to address this issue is to replace mutual information with other statistical measures. In the privacy literature, several measures with strong privacy guarantee have been proposed including Rényi maximal correlation [21,63,64], probability of correctly recovering [65,66], minimum mean-squared estimation error (MMSE) [67,68], $\chi^{2}$-information [69] (a special case of *f*-information to be described in Section 3), Arimoto’s and Sibson’s mutual information [61,70]—to be discussed in Section 3, maximal leakage [71], and local differential privacy [72]. All these measures ensure interpretable privacy guarantees. For instance, it is shown in [67,68] that if $\chi^{2}$-information between *Y* and *T* is sufficiently small, then no functions of *Y* can be efficiently reconstructed given *T*; thus providing an interpretable privacy guarantee.

Another limitation of mutual information is related to its estimation difficulty. It is known that mutual information can be estimated from *n* samples with the estimation error that scales as $O(\log n/\sqrt{n})$ [33]. However, as shown by Diaz et al. [34], the estimation error for most of the above measures scales as $O(1/\sqrt{n})$. Furthermore, the recently popular variational estimators for mutual information, typically implemented via deep learning methods [73,74,75], presents some fundamental limitations [76]: the variance of the estimator might grow exponentially with the ground truth mutual information and also the estimator might not satisfy basic properties of mutual information such as data processing inequality or additivity. McAllester and Stratos [77] showed that some of these limitations are inherent to a large family of mutual information estimators.

### 1.2. Notation

We use capital letters, e.g., *X*, for random variables and calligraphic letters for their alphabets, e.g., X. If *X* is distributed according to probability mass function (pmf) $P_{X}$, we write $X∼P_{X}$. Given two random variables *X* and *Y*, we write $P_{XY}$ and $P_{Y|X}$ as the joint distribution and the conditional distribution of *Y* given *X*. We also interchangeably refer to $P_{Y|X}$ as a channel from *X* to *Y*. We use H(X) to denote both entropy and differential entropy of *X*, i.e., we have

$$H\left(X\right)=-\sum_{x\in X}P_{X}\left(x\right)\log P_{X}\left(x\right)$$

if *X* is a discrete random variable taking values in X with probability mass function (pmf) $P_{X}$ and

$$H\left(X\right)=-\int \log f_{X}\left(x\right)\log f_{X}\left(x\right)dx,$$

where *X* is an absolutely continuous random variable with probability density function (pdf) $f_{X}$. If *X* is a binary random variable with $P_{X}\left(1\right)=p$, we write X∼Bernoulli(p). In this case, its entropy is called binary entropy function and denoted by $h_{b}\left(p\right)≔-p\log p-\left(1-p\right)\log\left(1-p\right)$. We use superscript G to describe a standard Gaussian random variable, i.e., $N^{G}∼N\left(0,1\right)$. Given two random variables *X* and *Y*, their (Shannon’s) mutual information is denoted by I(X;Y)≔H(Y)−H(Y|X). We let P(X) denote the set of all probability distributions on the set X. Given an arbitrary $Q_{X}\in P\left(X\right)$ and a channel $P_{Y|X}$, we let $Q_{X}P_{Y|X}$ denote the resulting output distribution on Y. For any a∈[0,1], we use $\bar{a}$ to denote 1−a and for any integer k∈N, [k]≔{1,2,…,k}.

Throughout the paper, we assume a pair of (discrete or continuous) random variables $\left(X,Y\right)∼P_{XY}$ are given with a fixed joint distribution $P_{XY}$, marginals $P_{X}$ and $P_{Y}$, and conditional distribution $P_{Y|X}$. We then use $Q_{X}\in P\left(X\right)$ to denote an arbitrary distribution with $Q_{Y}=Q_{X}P_{Y|X}\in P\left(Y\right)$.

## 2. Information Bottleneck and Privacy Funnel: Definitions and Functional Properties

In this section, we review the information bottleneck and its closely related functional, the privacy funnel. We then prove some analytical properties of these two functionals and develop a convex analytic approach which enables us to compute closed-form expressions for both these two functionals in some simple cases.

To precisely quantify the trade-off between these two conflicting goals, the IB optimization problem (2) was proposed [1]. Since any randomized function T=F(X) can be equivalently characterized by a conditional distribution, the optimization problem (2) can be instead expressed as

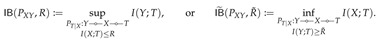
(4)
where *R* and $\tilde{R}$ denote the level of desired compression and informativeness, respectively. We use IB(R) and $\tilde{\mathrm{IB}}\left(\tilde{R}\right)$ to denote $\mathrm{IB}(P_{XY},R)$ and $\tilde{\mathrm{IB}}\left(P_{XY},\tilde{R}\right)$, respectively, when the joint distribution is clear from the context. Notice that if $\mathrm{IB}\left(P_{XY},R\right)=\tilde{R}$, then $\tilde{\mathrm{IB}}\left(P_{XY},\tilde{R}\right)=R$.

Now consider the setup where data *X* is required to be disclosed while maintaining the privacy of a sensitive attribute, represented by *Y*. This goal was formulated by PF in (3). As before, replacing randomized function T=F(X) with conditional distribution $P_{T|X}$, we can equivalently express (3) as

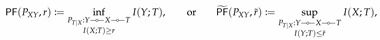
(5)
where $\tilde{r}$ and *r* denote the level of desired privacy and informativeness, respectively. The case $\tilde{r}=0$ is particularly interesting in practice and specifies perfect privacy, see e.g., [13,78]. As before, we write $\tilde{\mathrm{PF}}\left(\tilde{r}\right)$ and PF(r) for $\tilde{\mathrm{PF}}\left(P_{XY},\tilde{r}\right)$ and $\mathrm{PF}(P_{XY},r)$ when $P_{XY}$ is clear from the context.

The following properties of IB and PF follow directly from their definitions. The proof of this result (and any other results in this section) is given in Appendix A.

**Theorem** **1.** *For a given $P_{XY}$, the mappings IB(R) and PF(r) have the following properties:*

- *IB(0)=PF(0)=0.*
- *IB(R)=I(X;Y) for any R≥H(X) and PF(r)=I(X;Y) for r≥H(X).*
- *0≤IB(R)≤min{R,I(X;Y)} for any R≥0 and PF(r)≥max{r−H(X|Y),0} for any r≥0.*
- *R↦IB(R) is continuous, strictly increasing, and concave on the range (0,I(X;Y)).*
- *r↦PF(r) is continuous, strictly increasing, and convex on the range (0,I(X;Y)).*
- *If $P_{Y|X}\left(y|x\right)>0$ for all x∈X and y∈Y, then both R↦IB(R) and r↦PF(r) are continuously differentiable over (0,H(X)).*
- *$R\mapsto \frac{\mathrm{IB}(R)}{R}$ is non-increasing and $r\mapsto \frac{\mathrm{PF}(r)}{r}$ is non-decreasing.*
- *We have*

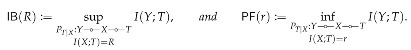

According to this theorem, we can always restrict both *R* and *r* in (4) and (5), respectively, to [0,H(X)] as IB(R)=PF(r)=I(X;Y) for all r,R≥H(X).

**Figure 1:**
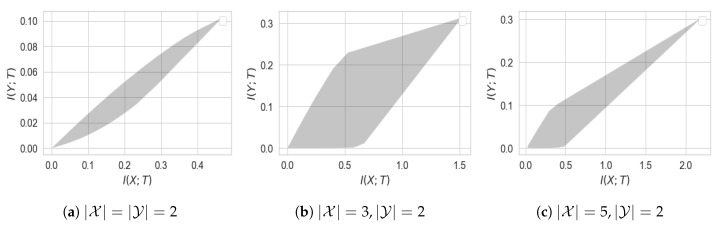
Examples of the set M, defined in (6). The upper and lower boundaries of this set correspond to information bottleneck (IB) and privacy funnel (PF), respectively. It is worth noting that, while IB(*R*) = 0 only at *R* = 0, PF(*r*) = 0 holds in general for *r* belonging to a non-trivial interval (only for |X| > 2). Moreover, note that in general neither upper nor lower boundaries are smooth. A sufficient condition for smoothness is $P_{X|Y}\left(y|x\right)$ > 0 (see Theorem 1), thus both IB and PF are smooth in the binary case.

Define $M=M\left(P_{XY}\right)\subset R^{2}$ as

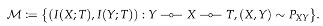
(6)
It can be directly verified that M is convex. According to this theorem, R↦IB(R) and r↦PF(r) correspond to the upper and lower boundary of M, respectively. The convexity of M then implies the concavity and convexity of IB and PF. Figure 1 illustrates the set M for the simple case of binary *X* and *Y*.

While both IB(0)=0 and PF(0)=0, their behavior in the neighborhood around zero might be completely different. As illustrated in Figure 1, IB(R)>0 for all R>0, whereas PF(r)=0 for $r\in [0,r_{0}]$ for some $r_{0}>0$. When such $r_{0}>0$ exists, we say perfect privacy occurs: there exists a variable *T* satisfying *Y* 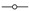 *X* 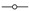 *T* such that I(Y;T)=0 while I(X;T)>0; making *T* a representation of *X* having perfect privacy (i.e., no information leakage about *Y*). A necessary and sufficient condition for the existence of such *T* is given in [21] (Lemma 10) and [13] (Theorem 3), described next.

**Theorem** **2** (Perfect privacy)**.** *Let $\left(X,Y\right)∼P_{XY}$ be given and $A\subset {\left[0,1\right]}^{\left|Y\right|}$ be the set of vectors $\left\{P_{Y|X}\left(\cdot |x\right),x\in X\right\}$. Then there exists $r_{0}>0$ such that PF(r)=0 for $r\in [0,r_{0}]$ if and only if vectors in A are linearly independent.*

In light of this theorem, we obtain that perfect privacy occurs if |X|>|Y|. It also follows from the theorem that for binary *X*, perfect privacy cannot occur (see Figure 1a).

Theorem 1 enables us to derive a simple bounds for IB and PF. Specifically, the facts that $\frac{\mathrm{PF}(r)}{r}$ is non-decreasing and $\frac{\mathrm{IB}(R)}{R}$ is non-increasing immediately result in the the following linear bounds.

**Theorem** **3** (Linear lower bound)**.** *For r,R∈(0,H(X)), we have*

(7) infQX∈P(X)QX≠PXDKL(QY∥PY)DKL(QX∥PX)≤PF(r)r≤I(X;Y)H(X)≤IB(R)R≤supQX∈P(X)QX≠PXDKL(QY∥PY)DKL(QX∥PX)≤1.

In light of this theorem, if PF(r)=r, then I(X;Y)=H(X), implying X=g(Y) for a deterministic function *g*. Conversely, if X=g(Y) then PF(r)=r because for all *T* forming the Markov relation *Y* 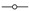 *g*(*Y*) 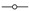 *T*, we have I(Y;T)=I(g(Y);T). On the other hand, we have IB(R)=R if and only if there exists a variable $T^{*}$ satisfying $I\left(X;T^{*}\right)=I\left(Y;T^{*}\right)$ and thus the following double Markov relations

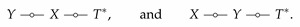

It can be verified (see [79] (Problem 16.25)) that this double Markov condition is equivalent to the existence of a pair of functions *f* and *g* such that f(X)=g(Y) and (*X,Y*) 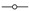 *f*(*X*) 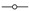 $T^{*}$. One special case of this setting, namely where *g* is an identity function, has been recently studied in details in [53] and will be reviewed in Section 2.5. Theorem 3 also enables us to characterize the “worst” joint distribution $P_{XY}$ with respect to IB and PF. As demonstrated in the following lemma, if $P_{Y|X}$ is an erasure channel then $\frac{\mathrm{PF}(r)}{r}=\frac{\mathrm{IB}(R)}{R}=\frac{I(X;Y)}{H(X)}$.

**Lemma** **1.**

- *Let $P_{XY}$ be such that Y=X∪{⊥}, $P_{Y|X}\left(x|x\right)=1-\delta$, and $P_{Y|X}\left(⊥|x\right)=\delta$ for some δ>0. Then*
  $$\frac{\mathrm{PF}(r)}{r}=\frac{\mathrm{IB}(R)}{R}=1-\delta .$$
- *Let $P_{XY}$ be such that X=Y∪{⊥}, $P_{X|Y}\left(y|y\right)=1-\delta$, and $P_{X|Y}\left(⊥|y\right)=\delta$ for some δ>0. Then*
  PF(r)=max{r−H(X|Y),0}.

The bounds in Theorem 3 hold for all *r* and *R* in the interval [0,H(X)]. We can, however, improve them when *r* and *R* are sufficiently small. Let ${\mathrm{PF}}^{\prime }\left(0\right)$ and ${\mathrm{IB}}^{\prime }\left(0\right)$ denote the slope of PF(·) and IB(·) at zero, i.e., ${\mathrm{PF}}^{\prime }\left(0\right)≔\lim_{r\to 0^{+}}\frac{\mathrm{PF}(r)}{r}$ and ${\mathrm{IB}}^{\prime }\left(0\right)≔\lim_{R\to 0^{+}}\frac{\mathrm{IB}(R)}{R}$.

**Theorem** **4.** *Given $\left(X,Y\right)∼P_{XY}$, we have*

infQX∈P(X)QX≠PXDKL(QY∥PY)DKL(QX∥PX)=PF′(0)≤minx∈X:PX(x)>0DKL(PY|X(·|x)∥PY(·))−logPX(x)≤maxx∈X:PX(x)>0DKL(PY|X(·|x)∥PY(·))−logPX(x)≤IB′(0)=supQX∈P(X)QX≠PXDKL(QY∥PY)DKL(QX∥PX).

This theorem provides the exact values of ${\mathrm{PF}}^{\prime }\left(0\right)$ and ${\mathrm{IB}}^{\prime }\left(0\right)$ and also simple bounds for them. While the exact expressions for ${\mathrm{PF}}^{\prime }\left(0\right)$ and ${\mathrm{IB}}^{\prime }\left(0\right)$ are usually difficult to compute, a simple plug-in estimator is proposed in [80] for ${\mathrm{IB}}^{\prime }\left(0\right)$. This estimator can be readily adapted to estimate ${\mathrm{PF}}^{\prime }\left(0\right)$. Theorem 4 reveals a profound connection between IB and the strong data processing inequality (SDPI) [81]. More precisely, thanks to the pioneering work of Anantharam et al. [82], it is known that the supremum of $\frac{D_{\mathrm{KL}}\left(Q_{Y}∥P_{Y}\right)}{D_{\mathrm{KL}}\left(Q_{X}∥P_{X}\right)}$ over all $Q_{X}\neq P_{X}$ is equal the supremum of $\frac{I(Y;T)}{I(X;T)}$ over all $P_{T|X}$ satisfying *Y* 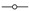 *X* 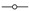 *T* and hence ${\mathrm{IB}}^{\prime }\left(0\right)$ specifies the strengthening of the data processing inequality of mutual information. This connection may open a new avenue for new theoretical results for IB, especially when *X* or *Y* are continuous random variables. In particular, the recent non-multiplicative SDPI results [27,83] seem insightful for this purpose.

In many practical cases, we might have *n* i.i.d. samples $\left(X_{1},Y_{1}\right),\ldots ,\left(X_{n},Y_{n}\right)$ of $\left(X,Y\right)∼P_{XY}$. We now study how IB behaves in *n*. Let $X^{n}≔\left(X_{1},\ldots ,X_{n}\right)$ and $Y^{n}≔\left(Y_{1},\ldots ,Y_{n}\right)$. Due to the i.i.d. assumption, we have $P_{X^{n}Y^{n}}\left(x^{n},y^{n}\right)=\prod_{i=1}^{n}P_{XY}\left(x_{i},y_{i}\right)$. This can also be described by independently feeding $X_{i}$, i∈[n], to channel $P_{Y|X}$ producing $Y_{i}$. The following theorem, demonstrated first in [3] (Theorem 2.4), gives a formula for IB in terms of *n*.

**Theorem** **5** (Additivity)**.** *We have*

$$\frac{1}{n}\mathrm{IB}\left(P_{X^{n}Y^{n}},nR\right)=\mathrm{IB}\left(P_{XY},R\right).$$

This theorem demonstrates that an optimal channel $P_{T^{n}|X^{n}}$ for i.i.d. samples $\left(X^{n},Y^{n}\right)∼P_{XY}$ is obtained by the Kronecker product of an optimal channel $P_{T|X}$ for $\left(X,Y\right)∼P_{XY}$. This, however, may not hold in general for PF, that is, we might have $\mathrm{PF}\left(P_{X^{n}Y^{n}},nr\right)<n\mathrm{PF}\left(P_{XY},r\right)$, see [13] (Proposition 1) for an example.

### 2.1. Gaussian IB and PF

In this section, we turn our attention to a special, yet important, case where $X=Y+\sigma N^{G}$, where σ>0 and $N^{G}∼N\left(0,1\right)$ is independent of *Y*. This setting subsumes the popular case of jointly Gaussian (X,Y) whose information bottleneck functional was computed in [84] for the vector case (i.e., (X,Y) are jointly Gaussian random vectors).

**Lemma** **2.** *Let ${\left\{Y_{i}\right\}}_{i=1}^{n}$ be n i.i.d. copies of $Y∼P_{Y}$ and $X_{i}=Y_{i}+\sigma N_{i}^{G}$ where $\left\{N_{i}^{G}\right\}$ are i.i.d samples of N(0,1) independent of Y. Then, we have*

$$\frac{1}{n}\mathrm{IB}\left(P_{X^{n}Y^{n}},nR\right)\leq H\left(X\right)-\frac{1}{2}\log\left[2\pi e\sigma^{2}+e^{2(H(Y)-R)}\right].$$

It is worth noting that this result was concurrently proved in [85]. The main technical tool in the proof of this lemma is a strong version of the entropy power inequality [25] (Theorem 2) which holds even if $X_{i}$, $Y_{i}$, and $N_{i}$ are random vectors (as opposed to scalar). Thus, one can readily generalize Lemma 2 to the vector case. Note that the upper bound established in this lemma holds without any assumptions on $P_{T|X}$. This upper bound provides a significantly simpler proof for the well-known fact that for the jointly Gaussian (X,Y), the optimal channel $P_{T|X}$ is Gaussian. This result was first proved in [26] and used in [84] to compute an expression of IB for the Gaussian case.

**Corollary** **1.** *If (X,Y) are jointly Gaussian with correlation coefficient ρ, then we have*

(8) $$\mathrm{IB}\left(R\right)=\frac{1}{2}\log\frac{1}{1-\rho^{2}+\rho^{2}e^{-2R}}.$$

*Moreover, the optimal channel $P_{T|X}$ is given by $P_{T|X}\left(\cdot |x\right)=N\left(0,{\tilde{\sigma }}^{2}\right)$ for ${\tilde{\sigma }}^{2}=\sigma_{Y}^{2}\frac{e^{-2R}}{\rho^{2}\left(1-e^{-2R}\right)}$ where $\sigma_{Y}^{2}$ is the variance of Y.*

In Lemma 2, we assumed that *X* is a Gaussian perturbation of *Y*. However, in some practical scenarios, we might have *Y* as a Gaussian perturbation of *X*. For instance, let *X* represent an image and *Y* be a feature of the image that can be perfectly obtained from a noisy observation of *X*. Then, the goal is to compress the image with a given compression rate while retaining maximal information about the feature. The following lemma, which is an immediate consequence of [27] (Theorem 1), gives an upper bound for IB in this case.

**Lemma** **3.** *Let $X^{n}$ be n i.i.d. copies of a random variable X satisfying $E[X^{2}]\leq 1$ and $Y_{i}$ be the result of passing $X_{i}$, i∈[n], through a Gaussian channel $Y=X+\sigma N^{G}$, where σ>0 and $N^{G}∼N\left(0,1\right)$ is independent of X. Then, we have*

(9) $$\frac{1}{n}\mathrm{IB}\left(P_{X^{n}Y^{n}},nR\right)\leq R-\Psi \left(R,\sigma \right),$$

*where*

(10) $$\Psi \left(R,\sigma \right)≔\max_{x\in [0,\frac{1}{2}]}2Q\left(\sqrt{\frac{1}{x\sigma^{2}}}\right)\left(R-h_{b}\left(x\right)-\frac{x}{2}\log\left(1+\frac{1}{x\sigma^{2}}\right)\right),$$

*$Q\left(t\right)≔\int_{t}^{\infty }\frac{1}{\sqrt{2\pi }}e^{-\frac{t^{2}}{2}}dt$ is the Gaussian complimentary CDF and $h_{b}\left(a\right)≔-a\log\left(a\right)-\left(1-a\right)\log\left(1-a\right)$ for a∈(0,1) is the binary entropy function. Moreover, we have*

(11) $$\frac{1}{n}\mathrm{IB}\left(P_{X^{n}Y^{n}},nR\right)\leq R-e^{-\frac{1}{R\sigma^{2}}\log\frac{1}{R}+\Theta \left(\log\frac{1}{R}\right)}.$$

**Figure 2:**
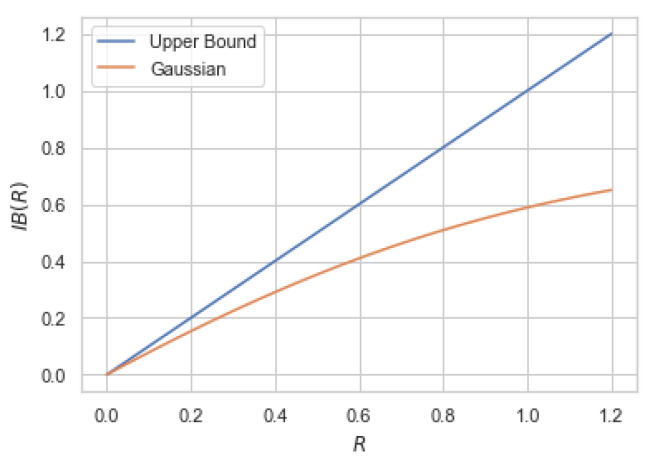
Comparison of (8), the exact value of IB for jointly Gaussian *X* and *Y* (i.e., $Y=X+\sigma N^{G}$ with *X* and $N^{G}$ being both standard Gaussian N(0,1)), with the general upper bound (9) for $\sigma^{2}=0.5$. It is worth noting that while the Gaussian IB converges to I(X;Y)≈0.8, the upper bound diverges.

Note that that Lemma 3 holds for any arbitrary *X* (provided that $E[X^{2}]\leq 1$) and hence (9) bounds information bottleneck functionals for a wide family of $P_{XY}$. However, the bound is loose in general for large values of *R*. For instance, if (X,Y) are jointly Gaussian (implying $Y=X+\sigma N^{G}$ for some σ>0), then the right-hand side of (9) does not reduce to (8). To show this, we numerically compute the upper bound (9) and compare it with the Gaussian information bottleneck (8) in Figure 2.

The privacy funnel functional is much less studied even for the simple case of jointly Gaussian. Solving the optimization in PF over $P_{T|X}$ without any assumptions is a difficult challenge. A natural assumption to make is that $P_{T|X}\left(\cdot |x\right)$ is Gaussian for each x∈X. This leads to the following variant of PF

$${\mathrm{PF}}^{G}\left(r\right)≔\underset{\begin{array}{c} \sigma \geq 0,\\ I(X;T_{\sigma })\geq r \end{array}}{\inf}I\left(Y;T_{\sigma }\right),$$

where

$$T_{\sigma }≔X+\sigma N^{G},$$

and $N^{G}∼N\left(0,1\right)$ is independent of *X*. This formulation is tractable and can be computed in closed form for jointly Gaussian (X,Y) as described in the following example.

**Example** **1.** *Let X and Y be jointly Gaussian with correlation coefficient ρ. First note that since mutual information is invariant to scaling, we may assume without loss of generality that both X and Y are zero mean and unit variance and hence we can write $X=\rho Y+\sqrt{1-\rho^{2}}M^{G}$ where $M^{G}∼N\left(0,1\right)$ is independent of Y. Consequently, we have*

(12) $$I\left(X;T_{\sigma }\right)=\frac{1}{2}\log\left(1+\frac{1}{\sigma^{2}}\right),$$

*and*

(13) $$I\left(Y;T_{\sigma }\right)=\frac{1}{2}\log\left(1+\frac{\rho^{2}}{1-\rho^{2}+\sigma^{2}}\right).$$

*In order to ensure $I(X;T_{\sigma })\geq r$, we must have $\sigma \leq {\left(e^{2r}-1\right)}^{-\frac{1}{2}}$. Plugging this choice of σ into (13), we obtain*

(14) $${\mathrm{PF}}^{G}\left(r\right)=\frac{1}{2}\log\left(\frac{1}{1-\rho^{2}\left(1-e^{-2r}\right)}\right).$$

This example indicates that for jointly Gaussian (X,Y), we have ${\mathrm{PF}}^{G}\left(r\right)=0$ if and only if r=0 (thus perfect privacy does not occur) and the constraint $I(X;T_{\sigma })=r$ is satisfied by a unique σ. These two properties in fact hold for all continuous variables *X* and *Y* with finite second moments as demonstrated in Lemma A1 in Appendix A. We use these properties to derive a second-order approximation of ${\mathrm{PF}}^{G}\left(r\right)$ when *r* is sufficiently small. For the following theorem, we use var(U) to denote the variance of the random variable *U* and $\mathrm{var}\left(U|V\right)≔E[{\left(U-E\left[U|V\right]\right)}^{2}|V]$. We use $\sigma_{X}^{2}=\mathrm{var}\left(X\right)$ for short.

**Theorem** **6.** *For any pair of continuous random variables (X,Y) with finite second moments, we have as r→0*

$${\mathrm{PF}}^{G}\left(r\right)=\eta \left(X,Y\right)r+\Delta \left(X,Y\right)r^{2}+o\left(r^{2}\right),$$

*where $\eta \left(X,Y\right)≔\frac{\mathrm{var}(E[X|Y])}{\sigma_{X}^{2}}$ and*

$$\Delta \left(X,Y\right)≔\frac{2}{\sigma_{X}^{4}}\left[E\left[{\mathrm{var}}^{2}\left(X|Y\right)\right]-\sigma_{X}^{2}E\left[\mathrm{var}\left(X|Y\right)\right]\right].$$

**Figure 3:**
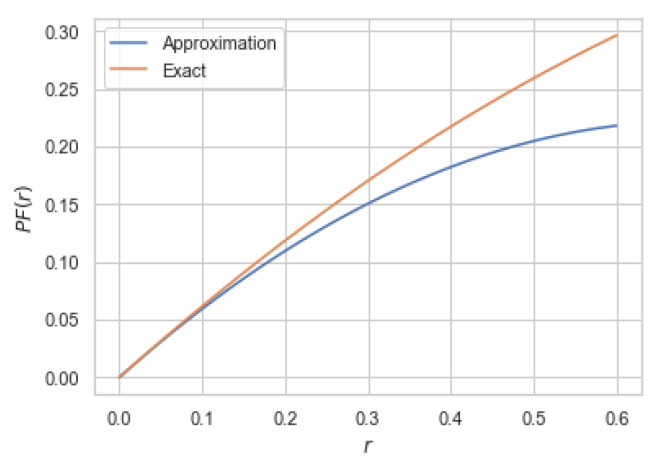
Second-order approximation of ${\mathrm{PF}}^{G}$ according to Theorem 6 for jointly Gaussian *X* and *Y* with correlation coefficient ρ=0.8. For this particular case, the exact expression of ${\mathrm{PF}}^{G}$ is computed in (14).

It is worth mentioning that the quantity η(X,Y) was first defined by Rényi [28] as an asymmetric measure of correlation between *X* and *Y*. In fact, it can be shown that $\eta \left(X,Y\right)=\sup_{f}\rho^{2}\left(X,f\left(Y\right)\right),$ where supremum is taken over all measurable functions *f* and ρ(·,·) denotes the correlation coefficient. As a simple illustration of Theorem 6, consider jointly Gaussian *X* and *Y* with correlation coefficient ρ for which ${\mathrm{PF}}^{G}$ was computed in Example 1. In this case, it can be easily verified that $\eta \left(X,Y\right)=\rho^{2}$ and $\Delta \left(X,Y\right)=-2\sigma_{X}^{2}\rho^{2}\left(1-\rho^{2}\right)$. Hence, for jointly Gaussian (X,Y) with correlation coefficient ρ and unit variance, we have ${\mathrm{PF}}^{G}\left(r\right)=\rho^{2}r-2\rho^{2}\left(1-\rho^{2}\right)r^{2}+o\left(r^{2}\right)$. In Figure 3, we compare the approximation given in Theorem 6 for this particular case.

### 2.2. Evaluation of IB and PF

The constrained optimization problems in the definitions of IB and PF are usually challenging to solve numerically due to the non-linearity in the constraints. In practice, however, both IB and PF are often approximated by their corresponding Lagrangian optimizations

(15) $$L_{\mathrm{IB}}\left(\beta \right)≔\underset{P_{T|X}}{\sup}I\left(Y;T\right)-\beta I\left(X;T\right)=H\left(Y\right)-\beta H\left(X\right)-\underset{P_{T|X}}{\inf}\left[H(Y|T)-\beta H(X|T)\right],$$

and

(16) $$L_{\mathrm{PF}}\left(\beta \right)≔\underset{P_{T|X}}{\inf}I\left(Y;T\right)-\beta I\left(X;T\right)=H\left(Y\right)-\beta H\left(X\right)-\underset{P_{T|X}}{\sup}\left[H(Y|T)-\beta H(X|T)\right],$$

where $\beta \in R_{+}$ is the Lagrangian multiplier that controls the tradeoff between compression and informativeness in for IB and the privacy and informativeness in PF. Notice that for the computation of $L_{\mathrm{IB}}$, we can assume, without loss of generality, that β∈[0,1] since otherwise the maximizer of (15) is trivial. It is worth noting that $L_{\mathrm{IB}}\left(\beta \right)$ and $L_{\mathrm{PF}}\left(\beta \right)$ in fact correspond to lines of slope β supporting M from above and below, thereby providing a new representation of M.

Let $\left(X^{\prime },Y^{\prime }\right)$ be a pair of random variables with $X^{\prime }∼Q_{X}$ for some $Q_{X}\in P\left(X\right)$ and $Y^{\prime }$ is the output of $P_{Y|X}$ when the input is $X^{\prime }$ (i.e., $Y^{\prime }∼Q_{X}P_{Y|X}$). Define

$$F_{\beta }\left(Q_{X}\right)≔H\left(Y^{\prime }\right)-\beta H\left(X^{\prime }\right).$$

This function, in general, is neither convex nor concave in $Q_{X}$. For instance, F(0) is concave and F(1) is convex in $P_{X}$. The lower convex envelope of $F_{\beta }\left(Q_{X}\right)$ is defined as the largest convex function smaller than $F_{\beta }\left(Q_{X}\right)$. Similarly, the upper concave envelope of $F_{\beta }\left(Q_{X}\right)$ is defined as the smallest concave function larger than $F_{\beta }\left(Q_{X}\right)$. Let $K_{\cup }\left[F_{\beta }\left(Q_{X}\right)\right]$ and $K_{\cap }\left[F_{\beta }\left(Q_{X}\right)\right]$ denote the lower convex and upper concave envelopes of $F_{\beta }\left(Q_{X}\right)$, respectively. If $F_{\beta }\left(Q_{X}\right)$ is convex at $P_{X}$, that is $K_{\cup }\left[F_{\beta }\left(Q_{X}\right)\right]{|}_{P_{X}}=F_{\beta }\left(P_{X}\right)$, then $F_{\beta }\left(Q_{X}\right)$ remains convex at $P_{X}$ for all $\beta^{\prime }\geq \beta$ because

$$\begin{array}{cc} K_{\cup }\left[F_{\beta^{\prime }}\left(Q_{X}\right)\right] & =K_{\cup }\left[F_{\beta }\left(Q_{X}\right)-\left(\beta^{\prime }-\beta \right)H\left(X^{\prime }\right)\right]\\ \; & \geq K_{\cup }\left[F_{\beta }\left(Q_{X}\right)\right]+K_{\cup }\left[-\left(\beta^{\prime }-\beta \right)H\left(X^{\prime }\right)\right]\\ \; & =K_{\cup }\left[F_{\beta }\left(Q_{X}\right)\right]-\left(\beta^{\prime }-\beta \right)H\left(X^{\prime }\right), \end{array}$$

where the last equality follows from the fact that $-\left(\beta^{\prime }-\beta \right)H\left(X\right)$ is convex. Hence, at $P_{X}$ we have

$$K_{\cup }\left[F_{\beta^{\prime }}\left(Q_{X}\right)\right]{|}_{P_{X}}\geq K_{\cup }\left[F_{\beta }\left(Q_{X}\right)\right]{|}_{P_{X}}-\left(\beta^{\prime }-\beta \right)H\left(X\right)=F_{\beta }\left(P_{X}\right)-\left(\beta^{\prime }-\beta \right)H\left(X\right)=F_{\beta^{\prime }}\left(P_{X}\right).$$

Analogously, if $F_{\beta }\left(Q_{X}\right)$ is concave at $P_{X}$, that is $K_{\cap }\left[F_{\beta }\left(Q_{X}\right)\right]{|}_{P_{X}}=F_{\beta }\left(P_{X}\right)$, then $F_{\beta }\left(Q_{X}\right)$ remains concave at $P_{X}$ for all $\beta^{\prime }\leq \beta$.

Notice that, according to (15) and (16), we can write

(17) $$L_{\mathrm{IB}}\left(\beta \right)=H\left(Y\right)-\beta H\left(X\right)-K_{\cup }\left[F_{\beta }\left(Q_{X}\right)\right]{|}_{P_{X}},$$

and

(18) $$L_{\mathrm{PF}}\left(\beta \right)=H\left(Y\right)-\beta H\left(X\right)-K_{\cap }\left[F_{\beta }\left(Q_{X}\right)\right]{|}_{P_{X}}.$$

In light of the above arguments, we can write

$$L_{\mathrm{IB}}\left(\beta \right)=0,$$

for all $\beta >\beta_{\mathrm{IB}}$ where $\beta_{\mathrm{IB}}$ is the smallest β such that $F_{\beta }\left(P_{X}\right)$ touches $K_{\cup }\left[F_{\beta }\left(Q_{X}\right)\right]$. Similarly,

$$L_{\mathrm{PF}}\left(\beta \right)=0,$$

for all $\beta <\beta_{\mathrm{PF}}$ where $\beta_{\mathrm{PF}}$ is the largest β such that $F_{\beta }\left(P_{X}\right)$ touches $K_{\cap }\left[F_{\beta }\left(Q_{X}\right)\right]$. In the following theorem, we show that $\beta_{\mathrm{IB}}$ and $\beta_{\mathrm{PF}}$ are given by the values of ${\mathrm{IB}}^{\prime }\left(0\right)$ and ${\mathrm{PF}}^{\prime }\left(0\right)$, respectively, given in Theorem 4. A similar formulae $\beta_{\mathrm{IB}}$ and $\beta_{\mathrm{PF}}$ were given in [86].

**Proposition** **1.** *We have,*

$$\beta_{\mathrm{IB}}=\underset{Q_{X}\neq P_{X}}{\sup}\frac{D_{\mathrm{KL}}\left(Q_{Y}∥P_{Y}\right)}{D_{\mathrm{KL}}\left(Q_{X}∥P_{X}\right)},$$

*and*

$$\beta_{\mathrm{PF}}=\underset{Q_{X}\neq P_{X}}{\inf}\frac{D_{\mathrm{KL}}\left(Q_{Y}∥P_{Y}\right)}{D_{\mathrm{KL}}\left(Q_{X}∥P_{X}\right)}.$$

Kim et al. [80] have recently proposed an efficient algorithm to estimate $\beta_{\mathrm{IB}}$ from samples of $P_{XY}$ involving a simple optimization problem. This algorithm can be readily adapted for estimating $\beta_{\mathrm{PF}}$. Proposition 1 implies that in optimizing the Lagrangians (17) and (18), we can restrict the Lagrange multiplier β, that is

(19) $$L_{\mathrm{IB}}\left(\beta \right)=H\left(Y\right)-\beta H\left(X\right)-K_{\cup }\left[F_{\beta }\left(Q_{X}\right)\right]{|}_{P_{X}},\;\mathrm{for}\;\beta \in \left[0,\beta_{\mathrm{IB}}\right],$$

and

(20) $$L_{\mathrm{PF}}\left(\beta \right)=H\left(Y\right)-\beta H\left(X\right)-K_{\cap }\left[F_{\beta }\left(Q_{X}\right)\right]{|}_{P_{X}},\;\mathrm{for}\;\beta \in \left[\beta_{\mathrm{PF}},\infty \right).$$

**Remark** **1.** *As demonstrated by Kolchinsky et al. [53], the boundary points 0 and $\beta_{\mathrm{IB}}$ are required for the computation of $L_{\mathrm{IB}}\left(\beta \right)$. In fact, when Y is a deterministic function of X, then* only *β=0 and $\beta =\beta_{\mathrm{IB}}$ are required to compute the IB and other values of β are vacuous. The same argument can also be used to justify the inclusion of $\beta_{\mathrm{PF}}$ in computing $L_{\mathrm{PF}}\left(\beta \right)$. Note also that since $F_{\beta }\left(Q_{X}\right)$ becomes convex for $\beta >\beta_{\mathrm{IB}}$, computing $K_{\cap }\left[F_{\beta }\left(Q_{X}\right)\right]$ becomes trivial for such values of β.*

**Remark** **2.** *Observe that the lower convex envelope of any function f can be obtained by taking Legendre-Fenchel transformation (aka. convex conjugate) twice. Hence, one can use the existing linear-time algorithms for approximating Legendre-Fenchel transformation (e.g., [87,88]) for approximating $K_{\cup }\left[F_{\beta }\left(Q_{X}\right)\right]$.*

Once $L_{\mathrm{IB}}\left(\beta \right)$ and $L_{\mathrm{PF}}\left(\beta \right)$ are computed, we can derive IB and PF via standard results in optimization (see [3] (Section IV) for more details):

(21) $$\mathrm{IB}\left(R\right)=\underset{\beta \in [0,\beta_{\mathrm{IB}}]}{\inf}\beta R+L_{\mathrm{IB}}\left(\beta \right),$$

and

(22) $$\mathrm{PF}\left(r\right)=\underset{\beta \in [\beta_{\mathrm{PF}},\infty ]}{\sup}\beta r+L_{\mathrm{PF}}\left(\beta \right).$$

Following the convex analysis approach outlined by Witsenhausen and Wyner [3], IB and PF can be directly computed from $L_{\mathrm{IB}}\left(\beta \right)$ and $L_{\mathrm{PF}}\left(\beta \right)$ by observing the following. Suppose for some β, $K_{\cup }\left[F_{\beta }\left(Q_{X}\right)\right]$ (resp. $K_{\cap }\left[F_{\beta }\left(Q_{X}\right)\right]$) at $P_{X}$ is obtained by a convex combination of points $F_{\beta }\left(Q^{i}\right)$, i∈[k] for some $Q^{1},\ldots ,Q^{k}$ in P(X), integer k≥2, and weights $\lambda_{i}\geq 0$ (with $\sum_{i}\lambda_{i}=1$). Then $\sum_{i}\lambda_{i}Q^{i}=P_{X}$, and $T^{*}$ with properties $P_{T^{*}}\left(i\right)=\lambda_{i}$ and $P_{X|T^{*}=i}=Q^{i}$ attains the minimum (resp. maximum) of H(Y|T)−βH(X|T). Hence, $\left(I\left(X;T^{*}\right),I\left(Y;T^{*}\right)\right)$ is a point on the upper (resp. lower) boundary of M; implying that $\mathrm{IB}\left(R\right)=I\left(Y;T^{*}\right)$ for $R=I(X;T^{*})$ (resp. $\mathrm{PF}\left(r\right)=I\left(Y;T^{*}\right)$ for $r=I(X;T^{*})$). If for some β, $K_{\cup }\left[F_{\beta }\left(Q_{X}\right)\right]$ at $P_{X}$ coincides with $F_{\beta }\left[P_{X}\right]$, then this corresponds to $L_{\mathrm{IB}}\left(\beta \right)=0$. The same holds for $K_{\cup }\left[F_{\beta }\left(Q_{X}\right)\right]$. Thus, all the information about the functional IB (resp. PF) is contained in the subset of the domain of $K_{\cup }\left[F_{\beta }\left(Q_{X}\right)\right]$ (resp. $K_{\cap }\left[F_{\beta }\left(Q_{X}\right)\right]$) over which it differs from $F_{\beta }\left(Q_{X}\right)$. We will revisit and generalize this approach later in Section 3.

We can now instantiate this for the binary symmetric case. Suppose *X* and *Y* are binary variables and $P_{Y|X}$ is binary symmetric channel with crossover probability δ, denoted by BSC(δ) and defined as

(23) $$\mathrm{BSC}\left(\delta \right)=\left[\begin{array}{cc} 1-\delta & \delta \\ \delta & 1-\delta \end{array}\right],$$

for some δ≥0. To describe the result in a compact fashion, we introduce the following notation: we let $h_{b}:\left[0,1\right]\to \left[0,1\right]$ denote the binary entropy function, i.e., $h_{b}\left(p\right)=-p\log p-\left(1-p\right)\log\left(1-p\right)$. Since this function is strictly increasing $\left[0,\frac{1}{2}\right]$, its inverse exists and is denoted by $h_{b}^{-1}:\left[0,1\right]\to \left[0,\frac{1}{2}\right]$. Moreover, a∗b≔a(1−b)+b(1−a) for a,b∈[0,1].

**Lemma** **4** (Mr. and Mrs. Gerber’s Lemma)**.** *For X∼Bernoulli(p) for $p\leq \frac{1}{2}$ and $P_{Y|X}=\mathrm{BSC}\left(\delta \right)$ for δ≥0, we have*

(24) $$\mathrm{IB}\left(R\right)=h_{b}\left(p*\delta \right)-h_{b}\left(\delta *h_{b}^{-1}\left(h_{b}\left(p\right)-R\right)\right),$$

*and*

(25) $$\mathrm{PF}\left(r\right)=h_{b}\left(p*\delta \right)-\alpha h_{b}\left(\delta *\frac{p}{z}\right)-\bar{\alpha }h_{b}\left(\delta \right),$$

*where $r=h_{b}\left(p\right)-\alpha h_{b}\left(\frac{p}{z}\right)$, $z=\max\left(\alpha ,2p\right)$, and α∈[0,1].*

**Figure 4:**
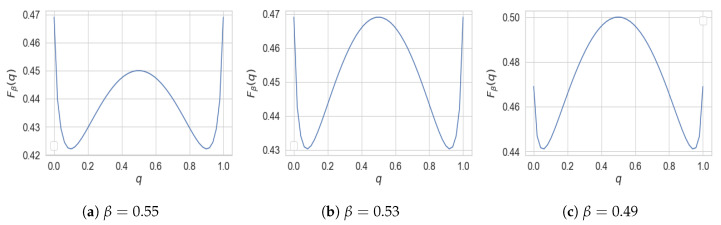
The mapping $q\mapsto F_{\beta }\left(q\right)=H\left(Y^{\prime }\right)-\beta H\left(X^{\prime }\right)$ where $X^{\prime }∼\mathrm{Bernoulli}\left(q\right)$ and $Y^{\prime }$ is the result of passing $X^{\prime }$ through BSC(0.1), see (26).

The result in (24) was proved by Wyner and Ziv [2] and is widely known as Mrs. Gerber’s Lemma in information theory. Due to the similarity, we refer to (25) as Mr. Gerber’s Lemma. As described above, to prove (24) and (25) it suffices to derive the convex and concave envelopes of the mapping $F_{\beta }:\left[0,1\right]\to R$ given by

(26) $$F_{\beta }\left(q\right)≔F_{\beta }\left(Q_{X}\right)=h_{b}\left(q*\delta \right)-\beta h_{b}\left(q\right),$$

where $q*\delta ≔q\bar{\delta }+\delta \bar{q}$ is the output distribution of BSC(δ) when the input distribution is Bernoulli(q) for some q∈(0,1). It can be verified that $\beta_{\mathrm{IB}}\leq {\left(1-2\delta \right)}^{2}$. This function is depicted in Figure 4 depending of the values of $\beta \leq {\left(1-2\delta \right)}^{2}$.

### 2.3. Operational Meaning of IB and PF

In this section, we illustrate several information-theoretic settings which shed light on the operational interpretation of both IB and PF. The operational interpretation of IB has recently been extensively studied in information-theoretic settings in [29,30]. In particular, it was shown that IB specifies the rate-distortion region of noisy source coding problem [19,89] under the logarithmic loss as the distortion measure and also the rate region of the lossless source coding with side information at the decoder [90]. Here, we state the former setting (as it will be useful for our subsequent analysis of cardinality bound) and also provide a new information-theoretic setting in which IB appears as the solution. Then, we describe another setting, the so-called dependence dilution, whose achievable rate region has an extreme point specified by PF. This in fact delineate an important difference between IB and PF: while IB describes the entire rate-region of an information-theoretic setup, PF specifies only a corner point of a rate region. Other information-theoretic settings related to IB and PF include CEO problem [91] and source coding for the Gray-Wyner network [92].

#### 2.3.1. Noisy Source Coding

Suppose Alice has access only to a noisy version *X* of a source of interest *Y*. She wishes to transmit a rate-constrained description from her observation (i.e., *X*) to Bob such that he can recover *Y* with small average distortion. More precisely, let $\left(X^{n},Y^{n}\right)$ be *n* i.i.d. samples of $\left(X,Y\right)∼P_{XY}$. Alice encodes her observation $X^{n}$ through an encoder $\phi :X^{n}\to \left\{1,\ldots ,K_{n}\right\}$ and sends $\phi (X^{n})$ to Bob. Upon receiving $\phi (X^{n})$, Bob reconstructs a “soft” estimate of $Y^{n}$ via a decoder $\psi :\left\{1,\ldots ,K_{n}\right\}\to {\hat{Y}}^{n}$ where $\hat{Y}=P\left(Y\right)$. That is, the reproduction sequence ${\hat{y}}^{n}$ consists of *n* probability measures on Y. For any source and reproduction sequences $y^{n}$ and ${\hat{y}}^{n}$, respectively, the distortion is defined as

$$d\left(y^{n},{\hat{y}}^{n}\right)≔\frac{1}{n}\sum_{i=1}^{n}d\left(y_{i},{\hat{y}}_{i}\right),$$

where

(27) $$d\left(y,\hat{y}\right)≔\log\frac{1}{\hat{y}\left(y\right)}.$$

We say that a pair of rate-distortion (R,D) is achievable if there exists a pair (ϕ,ψ) of encoder and decoder such that

(28) $$\underset{n\to \infty }{lim sup}E\left[d\left(Y^{n},\psi \left(\phi \left(X^{n}\right)\right)\right)\right]\leq D,\;\mathrm{and}\;\underset{n\to \infty }{lim sup}\frac{1}{n}\log K_{n}\leq R.$$

The noisy rate-distortion function $R^{\mathrm{noisy}}\left(D\right)$ for a given D≥0, is defined as the minimum rate R such that (R,D) is an achievable rate-distortion pair. This problem arises naturally in many data analytic problems. Some examples include feature selection of a high-dimensional dataset, clustering, and matrix completion. This problem was first studied by Dobrushin and Tsybakov [19], who showed that $R^{\mathrm{noisy}}\left(D\right)$ is analogous to the classical rate-distortion function

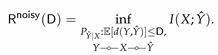
(29)
It can be easily verified that $E\left[d\left(Y,\hat{Y}\right)\right]=H\left(Y|\hat{Y}\right)$ and hence (after relabeling $\hat{Y}$ as *T*)

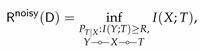
(30)
where R=H(Y)−D, which is equal to $\tilde{\mathrm{IB}}$ defined in (4). For more details in connection between noisy source coding and IB, the reader is referred to [29,30,91,93]. Notice that one can study an essentially identical problem where the distortion constraint (28) is replaced by

$$\lim_{n\to \infty }\frac{1}{n}I\left(Y^{n};\psi \left(\phi \left(X^{n}\right)\right)\right)]\geq R,\;\mathrm{and}\;\underset{n\to \infty }{lim sup}\frac{1}{n}\log K_{n}\leq R.$$

This problem is addressed in [94] for discrete alphabets X and Y and extended recently in [95] for any general alphabets.

#### 2.3.2. Test against Independence with Communication Constraint

As mentioned earlier, the connection between IB and noisy source coding, described above, was known and studied in [29,30]. Here, we provide a new information-theoretic setting which provides yet another operational meaning for IB. Given *n* i.i.d. samples $\left(X_{1},Y_{1}\right),\ldots ,\left(X_{n},Y_{n}\right)$ from joint distribution *Q*, we wish to test whether $X_{i}$ are independent of $Y_{i}$, that is, *Q* is a product distribution. This task is formulated by the following hypothesis test:

(31) $$\begin{array}{c} \begin{array}{cc} H_{0}: & \;\;Q=P_{XY},\\ H_{1}: & \;\;Q=P_{X}P_{Y}, \end{array} \end{array}$$

for a given joint distribution $P_{XY}$ with marginals $P_{X}$ and $P_{Y}$. Ahlswede and Csiszár [20] investigated this problem under a communication constraint: While *Y* observations (i.e., $Y_{1},\ldots ,Y_{n}$) are available, the *X* observations need to be compressed at rate *R*, that is, instead of $X^{n}$, only $\phi (X^{n})$ is present where $\phi :X^{n}\to \left\{1,\ldots ,K_{n}\right\}$ satisfies

$$\frac{1}{n}\log K_{n}\leq R.$$

For the type I error probability not exceeding a fixed ε∈(0,1), Ahlswede and Csiszár [20] derived the smallest possible type 2 error probability, defined as

βR(n,ε)=minϕ:Xn→[K]1nlogKn≤RminA⊂[Kn]×Yn(Pϕ(Xn)×PYn)(A):Pϕ(Xn)×Yn(A)≥1−ε.

The following gives the asymptotic expression of $\beta_{R}\left(n,\varepsilon \right)$ for every ε∈(0,1). For the proof, refer to [20] (Theorem 3).

**Theorem** **7** ([20])**.** *For every R≥0 and ε∈(0,1), we have*

$$\lim_{n\to \infty }-\frac{1}{n}\log\beta_{R}\left(n,\varepsilon \right)=\mathrm{IB}\left(R\right).$$

In light of this theorem, IB(R) specifies the exponential rate at which the type II error probability of the hypothesis test (31) decays as the number of samples increases.

#### 2.3.3. Dependence Dilution

Inspired by the problems of information amplification [96] and state masking [97], Asoodeh et al. [21] proposed the dependence dilution setup as follows. Consider a source sequences $X^{n}$ of *n* i.i.d. copies of $X∼P_{X}$. Alice observes the source $X^{n}$ and wishes to encode it via the encoder

$$f_{n}:X^{n}\to \left\{1,2,\ldots ,2^{nR}\right\},$$

for some R>0. The goal is to ensure that any user observing $f_{n}\left(X^{n}\right)$ can construct a list, of fixed size, of sequences in $X^{n}$ that contains likely candidates of the actual sequence $X^{n}$ while revealing negligible information about a correlated source $Y^{n}$. To formulate this goal, consider the decoder

$$g_{n}:\left\{1,2,\ldots ,2^{nR}\right\}\to 2^{X^{n}},$$

where $2^{X^{n}}$ denotes the power set of $X^{n}$. A *dependence dilution triple* $\left(R,\Gamma ,\Delta \right)\in R_{+}^{3}$ is said to be achievable if, for any δ>0, there exists a pair of encoder and decoder $\left(f_{n},g_{n}\right)$ such that for sufficiently large *n*

(32) $$\mathrm{Pr}\left(X^{n}\notin g_{n}\left(J\right)\right)<\delta ,$$

having fixed size $|g_{n}\left(J\right)|=2^{n(H(X)-\Gamma )},$ where $J=f_{n}\left(X^{n}\right)$ and simultaneously

(33) $$\frac{1}{n}I\left(Y^{n};J\right)\leq \Delta +\delta .$$

Notice that without side information *J*, the decoder can only construct a list of size $2^{nH(X)}$ which contains $X^{n}$ with probability close to one. However, after *J* is observed and the list $g_{n}\left(J\right)$ is formed, the decoder’s list size can be reduced to $2^{n(H(X)-\Gamma )}$ and thus reducing the uncertainty about $X^{n}$ by nΓ∈[0,nH(X)]. This observation can be formalized to show (see [96] for details) that the constraint (32) is equivalent to

(34) $$\frac{1}{n}I\left(X^{n};J\right)\geq \Gamma -\delta ,$$

which lower bounds the amount of information *J* carries about $X^{n}$. Built on this equivalent formulation, Asoodeh et al. [21] (Corollary 15) derived a necessary condition for the achievable dependence dilution triple.

**Theorem** **8** ([21])**.** *Any achievable dependence dilution triple (R,Γ,Δ) satisfies*

$$\left\{\begin{array}{cc} R & \geq \Gamma \\ \Gamma & \leq I(X;T)\\ \Delta & \geq I(Y;T)-I(X;T)+\Gamma , \end{array}\right.$$

*for some auxiliary random variable T satisfying *Y*
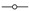
*X*
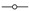
*T* and taking |T|≤|X|+1 values.*

According to this theorem, PF(Γ) specifies the best privacy performance of the dependence dilution setup for the maximum amplification rate Γ. While this informs the operational interpretation of PF, Theorem 8 only provides an outer bound for the set of achievable dependence dilution triple (R,Γ,Δ). It is, however, not clear that PF characterizes the rate region of an information-theoretic setup.

The fact that IB fully characterizes the rate-region of an source coding setup has an important consequence: the cardinality of the auxiliary random variable *T* in IB can be improved to |X| instead of |X|+1.

### 2.4. Cardinality Bound

Recall that in the definition of IB in (4), no assumption was imposed on the auxiliary random variable *T*. A straightforward application of Carathéodory-Fenchel-Eggleston theorem (see e.g., [98] (Section III) or [79] (Lemma 15.4)) reveals that IB is attained for *T* taking values in a set T with cardinality |T|≤|X|+1. Here, we improve this bound and show |T|≤|X| is sufficient.

**Theorem** **9.** *For any joint distribution $P_{XY}$ and R∈(0,H(X)], information bottleneck IB(R) is achieved by T taking at most |X| values.*

The proof of this theorem hinges on the operational characterization of IB as the lower boundary of the rate-distortion region of noisy source coding problem discussed in Section 2.3. Specifically, we first show that the extreme points of this region is achieved by *T* taking |X| values. We then make use of a property of the noisy source coding problem (namely, time-sharing) to argue that all points of this region (including the boundary points) can be attained by such *T*. It must be mentioned that this result was already claimed by Harremoës and Tishby in [99] without proof.

In many practical scenarios, feature *X* has a large alphabet. Hence, the bound |T|≤|X|, albeit optimal, still can make the information bottleneck function computationally intractable over large alphabets. However, label *Y* usually has a significantly smaller alphabet. While it is in general impossible to have a cardinality bound for *T* in terms of |Y|, one can consider approximating IB assuming *T* takes *N* values. The following result, recently proved by Hirche and Winter [100], is in this spirit.

**Theorem** **10** ([100])**.** *For any $\left(X,Y\right)∼P_{XY}$, we have*

IB(R,N)≤IB(R)≤IB(R,N)+δ(N),

*where $\delta \left(N\right)=4N^{-\frac{1}{\left|Y\right|}}\left[\log\frac{\left|Y\right|}{4}+\frac{1}{\left|Y\right|}\log N\right]$ and IB(R,N) denotes the information bottleneck functional (4) with the additional constraint that |T|≤N.*

**Figure 5:**
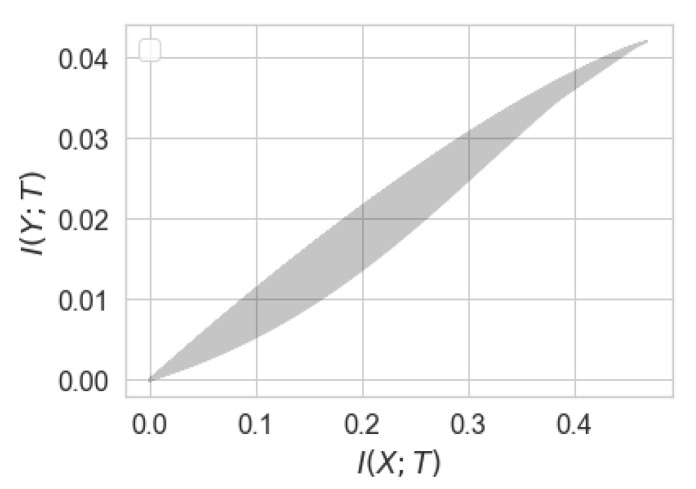
The set {(I(X;T),I(Y;T))} with $P_{X}=\mathrm{Bernoulli}\left(0.9\right)$, $P_{Y|X=0}=\left[0.9,0.1\right]$, $P_{Y|X=1}=\left[0.85,0.15\right]$, and *T* restricted to be binary. While the upper boundary of this set is concave, the lower boundary is not convex. This implies that, unlike IB, PF(r) cannot be attained by binary variables *T*.

Recall that, unlike PF, the graph of IB characterizes the rate region of a Shannon-theoretic coding problem (as illustrated in Section 2.3), and hence any boundary points can be constructed via time-sharing of extreme points of the rate region. This lack of operational characterization of PF translates into a worse cardinality bound than that of IB. In fact, for PF the cardinality bound |T|≤|X|+1 cannot be improved in general. To demonstrate this, we numerically solve the optimization in PF assuming that |T|=|X| when both *X* and *Y* are binary. As illustrated in Figure 5, this optimization does not lead to a convex function, and hence, cannot be equal to PF.

### 2.5. Deterministic Information Bottleneck

As mentioned earlier, IB formalizes an information-theoretic approach to clustering high-dimensional feature *X* into cluster labels *T* that preserve as much information about the label *Y* as possible. The clustering label is assigned by the soft operator $P_{T|X}$ that solves the IB formulation (4) according to the rule: X=x is likely assigned label T=t if $D_{\mathrm{KL}}\left(P_{Y|x}∥P_{Y|t}\right)$ is small where $P_{Y|t}=\sum_{x}P_{Y|x}P_{X|t}$. That is, clustering is assigned based on the similarity of conditional distributions. As in many practical scenarios, a hard clustering operator is preferred, Strouse and Schwab [31] suggested the following variant of IB, termed as deterministic information bottleneck dIB

(35) $$\mathrm{dIB}\left(P_{XY},R\right)≔\underset{\begin{array}{c} f:X\to T,\\ H(f(X))\leq R \end{array}}{\sup}I\left(Y;f\left(X\right)\right),$$

where the maximization is taken over all deterministic functions *f* whose range is a finite set T. Similarly, one can define

(36) $$\mathrm{dPF}\left(P_{XY},r\right)≔\underset{\begin{array}{c} f:X\to T,\\ H(f(X))\geq r \end{array}}{\inf}I\left(Y;f\left(X\right)\right).$$

One way to ensure that H(f(X))≤R for a deterministic function *f* is to restrict the cardinality of the range of *f*: if $f:X\to [e^{R}]$ then H(f(X)) is necessarily smaller than *R*. Using this insight, we derive a lower for $\mathrm{dIB}(P_{XY},R)$ in the following lemma.

**Lemma** **5.** *For any given $P_{XY}$, we have*

$$\mathrm{dIB}\left(P_{XY},R\right)\geq \frac{e^{R}-1}{\left|X\right|}I\left(X;Y\right),$$

*and*

$$\mathrm{dPF}\left(P_{XY},r\right)\leq \frac{e^{r}-1}{\left|X\right|}I\left(X;Y\right)+\mathrm{Pr}\left(X\geq e^{r}\right)\log\frac{1}{\mathrm{Pr}(X\geq e^{r})}.$$

Note that both *R* and *r* are smaller than H(X) and thus the multiplicative factors of I(X;Y) in the lemma are smaller than one. In light of this lemma, we can obtain

$$\frac{e^{R}-1}{\left|X\right|}I\left(X;Y\right)\leq \mathrm{IB}\left(R\right)\leq I\left(X;Y\right),$$

and

$$\mathrm{PF}\left(r\right)\leq \frac{e^{r}-1}{\left|X\right|}I\left(X;Y\right)+\mathrm{Pr}\left(X\geq e^{r}\right)\log\frac{1}{\mathrm{Pr}(X\geq e^{r})}.$$

In most of practical setups, |X| might be very large, making the above lower bound for IB vacuous. In the following lemma, we partially address this issue by deriving a bound independent of X when *Y* is binary.

**Lemma** **6.** *Let $P_{XY}$ be a joint distribution of arbitrary X and binary Y∼Bernoulli(q) for some q∈(0,1). Then, for any R≥log5 we have*

$$\mathrm{dIB}\left(P_{XY},R\right)\geq I\left(X;Y\right)-2\alpha h_{b}\left(\frac{I(X;Y)}{2\alpha (e^{R}-4)}\right),$$

*where $\alpha =\max\{\log\frac{1}{q},\log\frac{1}{1-q}\}$.*

## 3. Family of Bottleneck Problems

In this section, we introduce a family of bottleneck problems by extending IB and PF to a large family of statistical measures. Similar to IB and PF, these bottleneck problems are defined in terms of boundaries of a two-dimensional convex set induced by a joint distribution $P_{XY}$. Recall that $R\mapsto \mathrm{IB}(P_{XY},R)$ and $r\mapsto \mathrm{PF}(P_{XY},r)$ are the upper and lower boundary of the set M defined in (6) and expressed here again for convenience

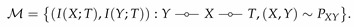
(37)
Since $P_{XY}$ is given, H(X) and H(Y) are fixed. Thus, in characterizing M it is sufficient to consider only H(X|T) and H(Y|T). To generalize IB and PF, we must therefore generalize H(X|T) and H(Y|T).

Given a joint distribution $P_{XY}$ and two non-negative real-valued functions $\Phi :P\left(X\right)\to R^{+}$ and $\Psi :P\left(Y\right)\to R^{+}$, we define

(38) $$\Phi \left(X|T\right)≔E\left[\Phi (P_{X|T})\right]=\sum_{t\in T}P_{T}\left(t\right)\Phi \left(P_{X|T=t}\right),$$

and

(39) $$\Psi \left(Y|T\right)≔E\left[\Psi (P_{Y|T})\right]=\sum_{t\in T}P_{T}\left(t\right)\Psi \left(P_{Y|T=t}\right).$$

When $X∼P_{X}$ and $Y∼P_{Y}$, we interchangeably write Φ(X) for $\Phi (P_{X})$ and Φ(Y) for $\Psi (P_{Y})$.

These definitions provide natural generalizations for Shannon’s entropy and mutual information. Moreover, as we discuss later in Section 3.2 and Section 3.3, it also can be specialized to represent a large family of popular information-theoretic and statistical measures. Examples include information and estimation theoretic quantities such as Arimoto’s conditional entropy of order α for $\Phi \left(Q_{X}\right)=\left|\right|Q_{X}{\left|\right|}_{\alpha }$, probability of correctly guessing for $\Phi \left(Q_{X}\right)=\left|\right|Q_{X}{\left|\right|}_{\infty }$, maximal correlation for binary case, and *f*-information for $\Phi (Q_{X})$ given by *f*-divergence. We are able to generate a family of bottleneck problems using different instantiations of Φ(X|T) and Ψ(Y|T) in place of mutual information in IB and PF. As we argue later, these problems better capture the essence of “informativeness” and “privacy”; thus providing analytical and interpretable guarantees similar in spirit to IB and PF.

Computing these bottleneck problems in general boils down to the following optimization problems

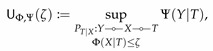
(40)
and

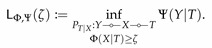
(41)
Consider the set

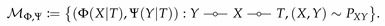
(42)
Note that if both Φ and Ψ are continuous (with respect to the total variation distance), then $M_{\Phi ,\Psi }$ is compact. Moreover, it can be easily verified that $M_{\Phi ,\Psi }$ is convex. Hence, its upper and lower boundaries are well-defined and are characterized by the graphs of $U_{\Phi ,\Psi }$ and $L_{\Phi ,\Psi }$, respectively. As mentioned earlier, these functional are instrumental for computing the general bottleneck problem later. Hence, before we delve into the examples of bottleneck problems, we extend the approach given in Section 2.2 to compute $U_{\Phi ,\Psi }$ and $L_{\Phi ,\Psi }$.

### 3.1. Evaluation of $U_{\Phi ,\Psi }$ and $L_{\Phi ,\Psi }$

Analogous to Section 2.2, we first introduce the Lagrangians of $U_{\Phi ,\Psi }$ and $L_{\Phi ,\Psi }$ as

(43) $$L_{\Phi ,\Psi }^{U}\left(\beta \right)≔\underset{P_{T|X}}{\sup}\Psi \left(Y|T\right)-\beta \Phi \left(X|T\right),$$

and

(44) $$L_{\Phi ,\Psi }^{L}\left(\beta \right)≔\underset{P_{T|X}}{\inf}\Psi \left(Y|T\right)-\beta \Phi \left(X|T\right),$$

where β≥0 is the Lagrange multiplier, respectively. Let $\left(X^{\prime },Y^{\prime }\right)$ be a pair of random variable with $X^{\prime }∼Q_{X}$ and $Y^{\prime }$ is the result of passing $X^{\prime }$ through the channel $P_{Y|X}$. Letting

(45) $$F_{\beta }^{\Phi ,\Psi }\left(Q_{X}\right)≔\Psi \left(Y^{\prime }\right)-\beta \Phi \left(X^{\prime }\right),$$

we obtain that

(46) $$L_{\Phi ,\Psi }^{U}\left(\beta \right)=K_{\cap }\left[F_{\beta }^{\Phi ,\Psi }\left(Q_{X}\right)\right]{|}_{P_{X}}\;\mathrm{and}\;L_{\Phi ,\Psi }^{L}\left(\beta \right)=K_{\cup }\left[F_{\beta }^{\Phi ,\Psi }\left(Q_{X}\right)\right]{|}_{P_{X}},$$

recalling that $K_{\cap }$ and $K_{\cup }$ are the upper concave and lower convex envelop operators. Once we compute $L_{\Phi ,\Psi }^{U}$ and $L_{\Phi ,\Psi }^{L}$ for all β≥0, we can use the standard results in optimizations theory (similar to (21) and (22)) to recover $U_{\Phi ,\Psi }$ and $L_{\Phi ,\Psi }$. However, we can instead extend the approach Witsenhausen and Wyner [3] described in Section 2.2. Suppose for some β, $K_{\cap }\left[F_{\beta }^{\Phi ,\Psi }\left(Q_{X}\right)\right]$ (resp. $K_{\cup }\left[F_{\beta }^{\Phi ,\Psi }\left(Q_{X}\right)\right]$) at $P_{X}$ is obtained by a convex combination of points $F_{\beta }^{\Phi ,\Psi }\left(Q^{i}\right)$, i∈[k] for some $Q^{1},\ldots ,Q^{k}$ in P(X), integer k≥2, and weights $\lambda_{i}\geq 0$ (with $\sum_{i}\lambda_{i}=1$). Then $\sum_{i}\lambda_{i}Q^{i}=P_{X}$, and $T^{*}$ with properties $P_{T^{*}}\left(i\right)=\lambda_{i}$ and $P_{X|T^{*}=i}=Q^{i}$ attains the maximum (resp. minimum) of Ψ(Y|T)−βΦ(X|T), implying that $\left(\Phi \left(X|T^{*}\right),\Psi \left(Y|T^{*}\right)\right)$ is a point on the upper (resp. lower) boundary of $M_{\Phi ,\Psi }$. Consequently, such $T^{*}$ satisfies $U_{\Phi ,\Psi }\left(\zeta \right)=\Psi \left(Y|T^{*}\right)$ for $\zeta =\Phi (X|T^{*})$ (resp. $L_{\Phi ,\Psi }\left(\zeta \right)=\Psi \left(Y|T^{*}\right)$ for $\zeta =\Phi (X|T^{*})$). The algorithm to compute $U_{\Phi ,\Psi }$ and $L_{\Phi ,\Psi }$ is then summarized in the following three steps:

- Construct the functional $F_{\beta }^{\Phi ,\Psi }\left(Q_{X}\right)≔\Psi \left(Y^{\prime }\right)-\beta \Phi \left(X^{\prime }\right)$ for $X^{\prime }∼Q_{X}$ and $Y^{\prime }∼Q_{X}P_{Y|X}$ and all $Q_{X}\in P\left(X\right)$ and β≥0.
- Compute $K_{\cap }\left[F^{\Phi ,\Psi }\left(Q_{X}\right)\right]{|}_{P_{X}}$ and $K_{\cup }\left[F^{\Phi ,\Psi }\left(Q_{X}\right)\right]{|}_{P_{X}}$ evaluated at $P_{X}$.
- If for distributions $Q^{1},\ldots ,Q^{k}$ in P(X) for some k≥1, we have $K_{\cap }\left[F^{\Phi ,\Psi }\left(Q_{X}\right)\right]{|}_{P_{X}}=\sum_{i=1}^{k}\lambda_{i}F^{\Phi ,\Psi }\left(Q^{i}\right)$ or $K_{\cup }\left[F^{\Phi ,\Psi }\left(Q_{X}\right)\right]{|}_{P_{X}}=\sum_{i=1}^{k}\lambda_{i}F^{\Phi ,\Psi }\left(Q^{i}\right)$ for some $\lambda_{i}\geq 0$ satisfying $\sum_{i=1}^{k}\lambda_{i}=1$, then then $P_{X|T=i}=Q_{i}$, i∈[k] and $P_{T}\left(i\right)=\lambda_{i}$ give the optimal $T^{*}$ in $U_{\Phi ,\Psi }$ and $L_{\Phi ,\Psi }$, respectively.

We will apply this approach to analytically compute $U_{\Phi ,\Psi }$ and $L_{\Phi ,\Psi }$ (and the corresponding bottleneck problems) for binary cases in the following sections.

### 3.2. Guessing Bottleneck Problems

Let $P_{XY}$ be given with marginals $P_{X}$ and $P_{Y}$ and the corresponding channel $P_{Y|X}$. Let also $Q_{X}\in P\left(X\right)$ be an arbitrary distribution on X and $Q_{Y}=Q_{X}P_{Y|X}$ be the output distribution of $P_{Y|X}$ when fed with $Q_{X}$. Any channel $P_{T|X}$, together with the Markov structure *Y* 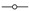 *X* 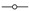 *T*, generates unique $P_{X|T}$ and $P_{Y|T}$. We need the following basic definition from statistics.

**Definition** **1.** *Let U be a discrete and V be an arbitrary random variables supported on U and V with |U|<∞, respectively. Then $P_{c}\left(U\right)$ the probability of correctly guessing U and $P_{c}\left(U|V\right)$ the probability of correctly guessing U given V are given by*

$$P_{c}\left(U\right)≔\max_{u\in U}P_{U}\left(u\right),$$

*and*

$$P_{c}\left(U|V\right)≔\max_{g}\mathrm{Pr}\left(U=g\left(V\right)\right)=E\left[\max_{u\in U}P_{U|V}\left(u|V\right)\right].$$

*Moreover, the multiplicative gain of the observation V in guessing U is defined as (the reason for ∞ in the notation becomes clear later)*

$$I_{\infty }\left(U;V\right)≔\log\frac{P_{c}\left(U|V\right)}{P_{c}\left(U\right)}.$$

As the names suggest, $P_{c}\left(U|V\right)$ and $P_{c}\left(U\right)$ characterize the optimal efficiency of guessing *U* with or without the observation *V*, respectively. Intuitively, $I_{\infty }\left(U;V\right)$ quantifies how useful the observation *V* is in estimating *U*: If it is small, then it means it is nearly as hard for an adversary observing *V* to guess *U* as it is without *V*. This observation motivates the use of $I_{\infty }\left(Y;T\right)$ as a measure of privacy in lieu of I(Y;T) in PF.

It is worth noting that $I_{\infty }\left(U;V\right)$ is not symmetric in general, i.e., $I_{\infty }\left(U;V\right)\neq I_{\infty }\left(V;U\right)$. Since observing *T* can only improve, we have $P_{c}\left(Y|T\right)\geq P_{c}\left(Y\right)$; thus $I_{\infty }\left(Y;T\right)\geq 0$. However, $I_{\infty }\left(Y;T\right)=0$ does not necessarily imply independent of *Y* and *T*; instead, it means *T* is useless in estimating *Y*. As an example, consider Y∼Bernoulli(p) and $P_{T|Y=0}=\mathrm{Bernoulli}\left(\delta \right)$ and $P_{T|Y=1}=\mathrm{Bernoulli}\left(\eta \right)$ with $\delta ,\eta \leq \frac{1}{2}<p$. Then $P_{c}\left(Y\right)=p$ and

$$P_{c}\left(Y|T\right)=\max\left\{\bar{\delta }\bar{p},\eta p\right\}+\bar{\eta }p.$$

Thus, if $\bar{\delta }\bar{p}\leq \eta p$, then $P_{c}\left(Y|T\right)=P_{c}\left(Y\right)$. This then implies that $I_{\infty }\left(Y;T\right)=0$ whereas *Y* and *T* are clearly dependent; i.e., I(Y;T)>0. While in general I(Y;T) and $I_{\infty }\left(Y;T\right)$ are not related, it can be shown that $I\left(Y;T\right)\leq I_{\infty }\left(Y;T\right)$ if *Y* is uniform (see [65] (Proposition 1)). Hence, only with this uniformity assumption, $I_{\infty }\left(Y;T\right)$ implies the independence.

Consider $\Psi \left(Q_{X}\right)=-\sum_{x\in X}Q_{X}\left(x\right)\log\left(Q_{X}\left(x\right)\right)$ and $\Psi \left(Q_{Y}\right)={∥Q_{Y}∥}_{\infty }$. Clearly, we have Φ(X|T)=H(X|T). Note that

(47) $$\Psi \left(Y|T\right)=\sum_{t\in T}P_{T}\left(t\right)||P_{Y|T=t}{\left|\right|}_{\infty }=P_{c}\left(Y|T\right),$$

thus both measures H(X|T) and $P_{c}\left(Y|T\right)$ are special cases of the models described in the previous section. In particular, we can define the corresponding $U_{\Phi ,\Psi }$ and $L_{\Phi ,\Psi }$. We will see later that I(X;T) and $P_{c}\left(Y|T\right)$ correspond to Arimoto’s mutual information of orders 1 and *∞*, respectively. Define

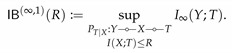
(48)
This bottleneck functional formulated an interpretable guarantee:

$${\mathrm{IB}}^{\left(\infty ,1\right)}\left(R\right)\mathrm{characterizes}\;\mathrm{the}\;\mathrm{best}\;\mathrm{error}\;\mathrm{probability}\;\mathrm{in}\;\mathrm{recovering}\;Y\;\;\mathrm{among}\;\mathrm{all}\;R-\mathrm{bit}\;\mathrm{summaries}\;\mathrm{of}\;X$$

Recall that the functional PF(r) aims at extracting maximum information of *X* while protecting privacy with respect to *Y*. Measuring the privacy in terms of $P_{c}\left(Y|T\right)$, this objective can be better formulated by

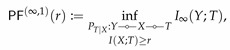
(49)
with the interpretable privacy guarantee:

$${\mathrm{PF}}^{\left(\infty ,1\right)}\left(r\right)\mathrm{characterizes}\mathrm{the}\mathrm{smallest}\mathrm{probability}\mathrm{of}\mathrm{revealing}\mathrm{private}\mathrm{feature}Y\;\mathrm{among}\mathrm{all}\mathrm{representations}\mathrm{of}X\mathrm{preserving}\mathrm{at}\mathrm{least}r\mathrm{bits}\mathrm{information}\mathrm{of}X$$

Notice that the variable *T* in the formulations of ${\mathrm{IB}}^{\left(\infty ,1\right)}$ and ${\mathrm{PF}}^{\left(\infty ,1\right)}$ takes values in a set T of arbitrary cardinality. However, a straightforward application of the Carathéodory-Fenchel-Eggleston theorem (see e.g., [79] (Lemma 15.4)) reveals that the cardinality of T can be restricted to |X|+1 without loss of generality. In the following lemma, we prove more basic properties of ${\mathrm{IB}}^{\left(\infty ,1\right)}$ and ${\mathrm{PF}}^{\left(\infty ,1\right)}$.

**Lemma** **7.** *For any $P_{XY}$ with Y supported on a finite set Y, we have*

- *${\mathrm{IB}}^{\left(\infty ,1\right)}\left(0\right)={\mathrm{PF}}^{\left(\infty ,1\right)}\left(0\right)=0$.*
- *${\mathrm{IB}}^{\left(\infty ,1\right)}\left(R\right)=I_{\infty }\left(X;Y\right)$ for any R≥H(X) and ${\mathrm{PF}}^{\left(\infty ,1\right)}\left(r\right)=I_{\infty }\left(X;Y\right)$ for r≥H(X).*
- *$R\mapsto \exp({\mathrm{IB}}^{\left(\infty ,1\right)}\left(R\right))$ is strictly increasing and concave on the range $\left(0,I_{\infty }\left(X;Y\right)\right)$.*
- *$r\mapsto \exp({\mathrm{PF}}^{\left(\infty ,1\right)}\left(r\right))$ is strictly increasing, and convex on the range $\left(0,I_{\infty }\left(X;Y\right)\right)$.*

The proof follows the same lines as Theorem 1 and hence omitted. Lemma 7 in particular implies that inequalities I(X;T)≤R and I(X;T)≥r in the definition of ${\mathrm{IB}}^{\left(\infty ,1\right)}$ and ${\mathrm{PF}}^{\left(\infty ,1\right)}$ can be replaced by I(X;T)=R and I(X;T)=r, respectively. It can be verified that $I^{\infty }$ satisfies the data-processing inequality, i.e., $I^{\infty }\left(Y;T\right)\leq I^{\infty }\left(Y;X\right)$ for the Markov chain *Y* 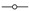 *X* 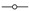 *T*. Hence, both ${\mathrm{IB}}^{\left(\infty ,1\right)}$ and ${\mathrm{PF}}^{\left(\infty ,1\right)}$ must be smaller than $I_{\infty }\left(Y;X\right)$. The properties listed in Lemma 7 enable us to derive a slightly tighter upper bound for ${\mathrm{PF}}^{\left(\infty ,1\right)}$ as demonstrated in the following.

**Lemma** **8.** *For any $P_{XY}$ with Y supported on a finite set Y, we have*

$${\mathrm{PF}}^{\left(\infty ,1\right)}\left(r\right)\leq \log\left[1+\frac{r}{H(X)}\left(e^{I_{\infty }\left(Y;X\right)}-1\right)\right],$$

*and*

$$\log\left[1+\frac{R}{H(X)}\left(e^{I_{\infty }\left(Y;X\right)}-1\right)\right]\leq {\mathrm{IB}}^{\left(\infty ,1\right)}\left(R\right)\leq I_{\infty }\left(Y;X\right).$$

The proof of this lemma (and any other results in this section) is given in Appendix B. This lemma shows that the gap between $I_{\infty }\left(Y;X\right)$ and ${\mathrm{IB}}^{\left(\infty ,1\right)}\left(R\right)$ when *R* is sufficiently close to H(X) behaves like

$$I_{\infty }\left(Y;X\right)-{\mathrm{IB}}^{\left(\infty ,1\right)}\left(R\right)\leq I_{\infty }\left(Y;X\right)-\log\left[1+\frac{R}{H(X)}\left(e^{I_{\infty }\left(Y;X\right)}-1\right)\right]\approx \left(1-e^{-I_{\infty }\left(Y;X\right)}\right)\left(1-\frac{R}{H(X)}\right).$$

Thus, ${\mathrm{IB}}^{\left(\infty ,1\right)}\left(R\right)$ approaches $I_{\infty }\left(Y;X\right)$ as R→H(X) at least linearly.

In the following theorem, we apply the technique delineated in Section 3.1 to derive closed form expressions for ${\mathrm{IB}}^{\left(\infty ,1\right)}$ and ${\mathrm{PF}}^{\left(\infty ,1\right)}$ for the binary symmetric case, thereby establishing similar results as Mr and Mrs. Gerber’s Lemma.

**Theorem** **11.** *For X∼Bernoulli(p) and $P_{Y|X}=\mathrm{BSC}\left(\delta \right)$ with $p,\delta \leq \frac{1}{2}$, we have*

(50) $${\mathrm{PF}}^{\left(\infty ,1\right)}\left(r\right)=\log\left[\frac{\bar{\delta }-\left(h_{b}\left(p\right)-r\right)\left(\frac{1}{2}-\delta \right)}{1-\delta *p}\right],$$

*and*

(51) $${\mathrm{IB}}^{\left(\infty ,1\right)}\left(R\right)=\log\left[\frac{1-\delta *h_{b}^{-1}\left(h_{b}\left(p\right)-R\right)}{1-\delta *p}\right],$$

*where $\bar{\delta }=1-\delta$.*

As described in Section 3.1, to compute ${\mathrm{IB}}^{\left(\infty ,1\right)}$ and ${\mathrm{PF}}^{\left(\infty ,1\right)}$ it suffices to derive the convex and concave envelopes of the mapping $F_{\beta }^{\left(\infty ,1\right)}\left(q\right)≔P_{c}\left(Y^{\prime }\right)+\beta H\left(X^{\prime }\right)$ where $X^{\prime }∼\mathrm{Bernoulli}\left(q\right)$ and $Y^{\prime }$ is the result of passing $X^{\prime }$ through BSC(δ), i.e., $Y^{\prime }∼\mathrm{Bernoulli}\left(\delta *q\right)$. In this case, $P_{c}\left(Y^{\prime }\right)=\max\left\{\delta *q,1-\delta *q\right\}$ and $F_{\beta }^{\left(\infty ,1\right)}$ can be expressed as

(52) $$q\mapsto F_{\beta }^{\left(\infty ,1\right)}\left(q\right)=\max\left\{\delta *q,1-\delta *q\right\}+\beta h_{b}\left(q\right).$$

**Figure 6:**
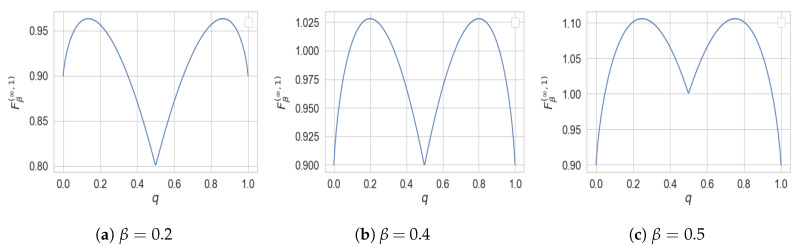
The mapping $q\mapsto F_{\beta }^{\left(\infty ,1\right)}\left(q\right)=P_{c}\left(Y^{\prime }\right)+\beta H\left(X^{\prime }\right)$ where $X^{\prime }∼\mathrm{Bernoulli}\left(q\right)$ and $Y^{\prime }∼\mathrm{Bernoulli}\left(q\right)\mathrm{BSC}(0.1)$.

This function is depicted in Figure 6.

The detailed derivation of convex and concave envelope of $F_{\beta }^{\left(\infty ,1\right)}$ is given in Appendix B. The proof of this theorem also reveals the following intuitive statements. If X∼Bernoulli(p) and $P_{Y|X}=\mathrm{BSC}\left(\delta \right)$, then among all random variables *T* satisfying *Y* 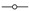 *X* 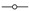 *T* and H(X|T)≤λ, the minimum $P_{c}\left(Y|T\right)$ is given by $\bar{\delta }-\lambda \left(0.5-\delta \right)$. Notice that, without any information constraint (i.e., λ=0), $P_{c}\left(Y|T\right)=P_{c}\left(Y|X\right)=\bar{\delta }$. Perhaps surprisingly, this shows that the mutual information constraint has a linear effect on the privacy of *Y*. Similarly, to prove (51), we show that among all *R*-bit representations *T* of *X*, the best achievable accuracy $P_{c}\left(Y|T\right)$ is given by $1-\delta *h_{b}^{-1}\left(h_{b}\left(p\right)-R\right)$. This can be proved by combining Mrs. Gerber’s Lemma (cf. Lemma 4) and Fano’s inequality as follows. For all *T* such that H(X|T)≥λ, the minimum of H(Y|T) is given by $h_{b}\left(\delta *h_{b}^{-1}\left(\lambda \right)\right)$. Since by Fano’s inequality, $H\left(Y|T\right)\leq h_{b}\left(1-P_{c}\left(Y|T\right)\right)$, we obtain $\delta *h_{b}^{-1}\left(\lambda \right)\leq 1-P_{c}\left(Y|T\right)$ which leads to the same result as above. Nevertheless, in Appendix B we give another proof based on the discussion of Section 3.1.

### 3.3. Arimoto Bottleneck Problems

The bottleneck framework proposed in the last section benefited from interpretable guarantees brought forth by the quantity $I_{\infty }$. In this section, we define a parametric family of statistical quantities, the so-called Arimoto’s mutual information, which includes both Shannon’s mutual information and $I_{\infty }$ as extreme cases.

**Definition** **2** ([22])**.** *Let $U∼P_{U}$ and $V∼P_{V}$ be two random variables supported over finite sets U and V, respectively. Their Arimoto’s mutual information of order α>1 is defined as*

(53) $$I_{\alpha }\left(U;V\right)=H_{\alpha }\left(U\right)-H_{\alpha }\left(U|V\right),$$

*where*

(54) $$H_{\alpha }\left(U\right)≔\frac{\alpha }{1-\alpha }\log||P_{U}{\left|\right|}_{\alpha },$$

*is the Rényi entropy of order α and*

(55) $$H_{\alpha }\left(U|V\right)≔\frac{\alpha }{1-\alpha }\log\sum_{v\in V}P_{V}\left(v\right)||P_{U|V=v}{\left|\right|}_{\alpha },$$

*is the Arimoto’s conditional entropy of order α.*

By continuous extension, one can define I−α(U;V) for α=1 and α=∞ as I(U;V) and $I_{\infty }\left(U;V\right)$, respectively. That is,

(56) $$\lim_{\alpha \to 1^{+}}I_{\alpha }\left(U;V\right)=I\left(U;V\right),\;\mathrm{and}\;\lim_{\alpha \to \infty }I_{\alpha }\left(U;V\right)=I_{\infty }\left(U;V\right).$$

Arimoto’s mutual information was first introduced by Arimoto [22] and then later revisited by Liese and Vajda in [101] and more recently by Verdú in [102]. More in-depth analysis and properties of $I_{\alpha }$ can be found in [103]. It is shown in [71] (Lemma 1) that $I_{\alpha }\left(U;V\right)$ for α∈[1,∞] quantifies the minimum loss in recovering *U* given *V* where the loss is measured in terms of the so-called α-loss. This loss function reduces to logarithmic loss (27) and $P_{c}\left(U|V\right)$ for α=1 and α=∞, respectively. This sheds light on the utility and/or privacy guarantee promised by a constraint on Arimoto’s mutual information. It is now natural to use $I_{\alpha }$ for defining a family of bottleneck problems.

**Definition** **3.** *Given a pair of random variables $\left(X,Y\right)∼P_{XY}$ over finite sets X and Y and α,γ∈[1,∞], we define ${\mathrm{IB}}^{\left(\alpha ,\gamma \right)}$ and ${\mathrm{PF}}^{\left(\alpha ,\gamma \right)}$ as*

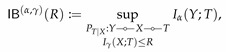
(57)
*and*

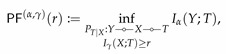
(58)

Of course, ${\mathrm{IB}}^{\left(1,1\right)}\left(R\right)=\mathrm{IB}\left(R\right)$ and ${\mathrm{PF}}^{\left(1,1\right)}\left(r\right)=\mathrm{PF}\left(r\right)$. It is known that Arimoto’s mutual information satisfies the data-processing inequality [103] (Corollary 1), i.e., $I_{\alpha }\left(Y;T\right)\leq I_{\alpha }\left(Y;X\right)$ for the Markov chain *Y* 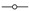 *X* 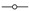 *T*. On the other hand, $I_{\gamma }\left(X;T\right)\leq H_{\gamma }\left(X\right)$. Thus, both ${\mathrm{IB}}^{\left(\alpha ,\gamma \right)}\left(R\right)$ and ${\mathrm{PF}}^{\left(\alpha ,\gamma \right)}\left(r\right)$ equal $I_{\alpha }\left(Y;X\right)$ for $R,r\geq H_{\gamma }\left(X\right)$. Note also that $H_{\alpha }\left(Y|T\right)=\frac{\alpha }{1-\alpha }\log\Psi \left(Y|T\right)$ where Ψ(Y|T) (see (39)) corresponding to the function $\Psi \left(Q_{Y}\right)=\left|\right|Q_{Y}{\left|\right|}_{\alpha }$. Consequently, ${\mathrm{IB}}^{\left(\alpha ,\gamma \right)}$ and ${\mathrm{PF}}^{\left(\alpha ,\gamma \right)}$ are characterized by the lower and upper boundary of $M_{\Phi ,\Psi }$, defined in (37), with respect to $\Phi \left(Q_{X}\right)=\left|\right|Q_{X}{\left|\right|}_{\gamma }$ and $\Psi \left(Q_{Y}\right)=\left|\right|Q_{Y}{\left|\right|}_{\alpha }$. Specifically, we have

(59) $${\mathrm{IB}}^{\left(\alpha ,\gamma \right)}\left(R\right)=H_{\alpha }\left(Y\right)+\frac{\alpha }{\alpha -1}\log U_{\Phi ,\Psi }\left(\zeta \right),$$

where $\zeta =e^{-\left(1-\frac{1}{\gamma }\right)\left(H_{\gamma }\left(X\right)-R\right)}$, and

(60) $${\mathrm{PF}}^{\left(\alpha ,\gamma \right)}\left(r\right)=H_{\alpha }\left(Y\right)+\frac{\alpha }{\alpha -1}\log L_{\Phi ,\Psi }\left(\zeta \right),$$

where $\zeta =e^{-\left(1-\frac{1}{\gamma }\right)\left(H_{\gamma }\left(X\right)-r\right)}$ and $\Phi \left(Q_{X}\right)={∥Q_{X}∥}_{\gamma }$ and $\Psi \left(Q_{Y}\right)={∥Q_{Y}∥}_{\alpha }$. This paves the way to apply the technique described in Section 2.2 to compute ${\mathrm{IB}}^{\left(\alpha ,\gamma \right)}$ and ${\mathrm{PF}}^{\left(\alpha ,\gamma \right)}$. Doing so requires the upper concave and lower convex envelope of the mapping $Q_{X}\mapsto ∥Q_{Y}{∥}_{\alpha }-\beta {∥Q_{X}∥}_{\gamma }$ for some β≥0, where $Q_{Y}∼Q_{X}P_{Y|X}$. In the following theorem, we drive these envelopes and give closed form expressions for ${\mathrm{IB}}^{\left(\alpha ,\gamma \right)}$ and ${\mathrm{PF}}^{\left(\alpha ,\gamma \right)}$ for a special case where α=γ≥2.

**Theorem** **12.** *Let X∼Bernoulli(p) and $P_{Y|X}=\mathrm{BSC}\left(\delta \right)$ with $p,\delta \leq \frac{1}{2}$. We have for α≥2*

$${\mathrm{PF}}^{\left(\alpha ,\alpha \right)}\left(r\right)=\frac{\alpha }{1-\alpha }\log\left[\frac{{∥p*\delta ∥}_{\alpha }}{{∥q*\delta ∥}_{\alpha }}\right],$$

*where ${∥a∥}_{\alpha }≔{∥\left[a,\bar{a}\right]∥}_{\alpha }$ for a∈[0,1] and q≤p solves*

$$\frac{\alpha }{1-\alpha }\log\left[\frac{{∥p∥}_{\alpha }}{{∥q∥}_{\alpha }}\right]=r.$$

*Moreover,*

$${\mathrm{IB}}^{\left(\alpha ,\alpha \right)}\left(R\right)=\frac{\alpha }{\alpha -1}\log\left[\frac{\bar{\lambda }{∥\delta ∥}_{\alpha }+\lambda {∥\frac{q}{z}*\delta ∥}_{\alpha }}{{∥p*\alpha ∥}_{\alpha }}\right],$$

*where z=max{2p,λ} and λ∈[0,1] solves*

$$\frac{\alpha }{\alpha -1}\log\frac{\bar{\lambda }+\lambda {∥\frac{p}{z}∥}_{\alpha }}{{∥p∥}_{\alpha }}=R.$$

**Figure 7:**
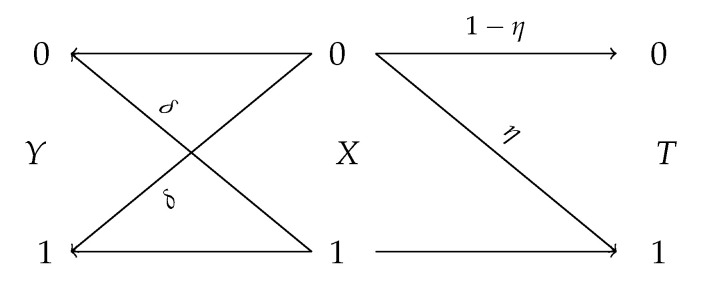
The structure of the optimal $P_{T|X}$ for ${\mathrm{PF}}^{\left(\infty ,\infty \right)}$ when $P_{Y|X}=\mathrm{BSC}\left(\delta \right)$ and X∼Bernoulli(p) with $\delta ,p\in [0,\frac{1}{2}]$. If the accuracy constraint is $P_{c}\left(X|T\right)\geq \lambda$ (or equivalently $I_{\infty }\left(X|T\right)\geq \log\frac{\lambda }{\bar{p}}$), then the parameter of optimal $P_{T|X}$ is given by $\eta =\frac{\bar{\lambda }}{\bar{p}}$, leading to $P_{c}\left(Y|T\right)=\delta *\lambda$.

By letting α→∞, this theorem indicates that for *X* and *Y* connected through BSC(δ) and all variables *T* forming *Y* 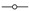 *X* 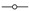 *T*, we have

(61) $$P_{c}\left(X|T\right)\geq \lambda \;⟹\;P_{c}\left(Y|T\right)\geq \delta *\lambda ,$$

which can be shown to be achieved $T^{*}$ generated by the following channel (see Figure 7)

(62) $$P_{T^{*}|X}=\left[\begin{array}{cc} \frac{\lambda -p}{\bar{p}} & \frac{\bar{\lambda }}{\bar{p}}\\ 0 & 1 \end{array}\right].$$

Note that, by assumption, $p\leq \frac{1}{2}$, and hence the event {X=1} is less likely than {X=0}. Therefore, (61) demonstrates that to ensure correct recoverability of *X* with probability at lest λ, the most private approach (with respect to *Y*) is to obfuscate the higher-likely event {X=0} with probability $\frac{\bar{\lambda }}{\bar{p}}$. As demonstrated in (61) the optimal privacy guarantee is linear in the utility parameter in the binary symmetric case. This is in fact a special case of the larger result recently proved in [65] (Theorem 1): the infimum of $P_{c}\left(Y|T\right)$ over all variables *T* such that $P_{c}\left(X|T\right)\geq \lambda$ is piece-wise linear in λ, on equivalently, the mapping $e^{r}\mapsto \exp\left({\mathrm{PF}}^{\left(\infty ,\infty \right)}\left(r\right)\right)$ is piece-wise linear.

Computing ${\mathrm{PF}}^{\left(\alpha ,\gamma \right)}$ analytically for every α,γ>1 seems to be challenging, however, the following lemma provides bounds for ${\mathrm{PF}}^{\left(\alpha ,\gamma \right)}$ and ${\mathrm{IB}}^{\left(\alpha ,\gamma \right)}$ in terms of ${\mathrm{PF}}^{\left(\infty ,\infty \right)}$ and ${\mathrm{IB}}^{\left(\infty ,\infty \right)}$, respectively.

**Lemma** **9.** *For any pair of random variables (X,Y) over finite alphabets and α,γ>1, we have*

$$\frac{\alpha }{\alpha -1}{\mathrm{PF}}^{\left(\infty ,\infty \right)}\left(f\left(r\right)\right)-\frac{\alpha }{\alpha -1}H_{\infty }\left(Y\right)+H_{\alpha }\left(Y\right)\leq {\mathrm{PF}}^{\left(\alpha ,\gamma \right)}\left(r\right)\leq {\mathrm{PF}}^{\left(\infty ,\infty \right)}\left(g\left(r\right)\right)+H_{\alpha }\left(Y\right)-H_{\infty }\left(Y\right),$$

*and*

$$\frac{\alpha }{\alpha -1}{\mathrm{IB}}^{\left(\infty ,\infty \right)}\left(f\left(R\right)\right)-\frac{\alpha }{\alpha -1}H_{\infty }\left(Y\right)+H_{\alpha }\left(Y\right)\leq {\mathrm{IB}}^{\left(\alpha ,\gamma \right)}\left(R\right)\leq {\mathrm{IB}}^{\left(\infty ,\infty \right)}\left(g\left(R\right)\right)+H_{\alpha }\left(Y\right)-H_{\infty }\left(Y\right),$$

*where $f\left(a\right)=\max\{a-H_{\gamma }\left(X\right)+H_{\infty }\left(X\right),0\}$ and $g\left(b\right)=\frac{\gamma -1}{\gamma }b+H_{\infty }\left(X\right)-\frac{\gamma -1}{\gamma }H_{\gamma }\left(X\right)$.*

The previous lemma can be directly applied to derive upper and lower bounds for ${\mathrm{PF}}^{\left(\alpha ,\gamma \right)}$ and ${\mathrm{IB}}^{\left(\alpha ,\gamma \right)}$ given ${\mathrm{PF}}^{\left(\infty ,\infty \right)}$ and ${\mathrm{IB}}^{\left(\infty ,\infty \right)}$.

### 3.4. f-Bottleneck Problems

In this section, we describe another instantiation of the general framework introduced in terms of functions Φ and Ψ that enjoys interpretable estimation-theoretic guarantee.

**Definition** **4.** *Let f:(0,∞)→R be a convex function with f(1)=0. Furthermore, let U and V be two real-valued random variables supported over U and V, respectively. Their f-information is defined by*

(63) $$I_{f}\left(U;V\right)≔D_{f}\left(P_{UV}∥P_{U}P_{V}\right),$$

*where $D_{f}\left(\cdot ∥\cdot \right)$ is the f-divergence [104] between distributions and defined as*

$$D_{f}\left(P∥Q\right)≔E_{Q}\left[f\left(\frac{dP}{dQ}\right)\right].$$

Due to convexity of *f*, we have $D_{f}\left(P∥Q\right)\geq f\left(1\right)=0$ and hence *f*-information is always non-negative. If, furthermore, *f* is strictly convex at 1, then equality holds if and only P=Q. Csiszár introduced *f*-divergence in [104] and applied it to several problems in statistics and information theory. More recent developments about the properties of *f*-divergence and *f*-information can be found in [23] and the references therein. Any convex function *f* with the property f(1)=0 results in an *f*-information. Popular examples include f(t)=tlogt corresponding to Shannon’s mutual information, f(t)=|t−1| corresponding to *T*-information [83], and also $f\left(t\right)=t^{2}-1$ corresponding to $\chi^{2}$-information [69] for. It is worth mentioning that if we allow α to be in (0,1) in Definition 2 (similar to [101]), then the resulting Arimoto’s mutual information can be shown to be an *f*-information in the binary case for a certain function *f*, see [101] (Theorem 8).

Let $\left(X,Y\right)∼P_{XY}$ be given with marginals $P_{X}$ and $P_{Y}$. Consider functions Φ and Ψ on P(X) and P(Y) defined as

$$\Phi \left(Q_{X}\right)≔D_{f}\left(Q_{X}∥P_{X}\right)\;\mathrm{and}\;\Psi \left(Q_{Y}\right)≔D_{f}\left(Q_{Y}∥P_{Y}\right).$$

Given a conditional distribution $P_{T|X}$, it is easy to verify that $\Phi \left(X|T\right)=I_{f}\left(X;T\right)$ and $\Psi \left(Y|T\right)=I_{f}\left(Y;T\right)$. This in turn implies that *f*-information can be utilized in (40) and (41) to define general bottleneck: Let f:(0,∞)→R and g:(0,∞)→R be two convex functions satisfying f(1)=g(1)=0. Then we define

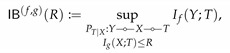
(64)
and

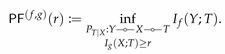
(65)

In light of the discussion in Section 3.1, the optimization problems in ${\mathrm{IB}}^{\left(f,g\right)}$ and ${\mathrm{IB}}^{\left(f,g\right)}$ can be analytically solved by determining the upper concave and lower convex envelope of the mapping

(66) $$Q_{X}\mapsto F_{\beta }^{\left(f,g\right)}≔D_{f}\left(Q_{Y}∥P_{Y}\right)-\beta D_{g}\left(Q_{X}∥P_{X}\right),$$

where β≥0 is the Lagrange multiplier and $Q_{Y}=Q_{X}P_{Y|X}$.

Consider the function $f_{\alpha }\left(t\right)=\frac{t^{\alpha }-1}{\alpha -1}$ with α∈(1,∞)∪(1,∞). The corresponding *f*-divergence is sometimes called Hellinger divergence of order α, see e.g., [105]. Note that Hellinger divergence of order 2 reduces to $\chi^{2}$-divergence. Calmon et al. [68] and Asoodeh et al. [67] showed that if $I_{f_{2}}\left(Y;T\right)\leq \varepsilon$ for some ε∈(0,1), then the minimum mean-squared error (MMSE) of reconstructing any zero-mean unit-variance function of *Y* given *T* is lower bounded by 1−ε, i.e., no function of *Y* can be reconstructed with small MMSE given an observation of *T*. This result serves a natural justification for $I_{f_{2}}$ as an operational measure of both privacy and utility in a bottleneck problem.

Unfortunately, our approach described in Section 3.1 cannot be used to compute ${\mathrm{IB}}^{\left(f_{2},f_{2}\right)}$ or ${\mathrm{PF}}^{\left(f_{2},f_{2}\right)}$ in the binary symmetric case. The difficulty lies in the fact that the function $F_{\beta }^{f_{2},f_{2}}$, defined in (66), for the binary symmetric case is either convex or concave on its entire domain depending on the value of β. Nevertheless, one can consider Hellinger divergence of order α with α≠2 and then apply our approach to compute ${\mathrm{IB}}^{\left(f_{\alpha },f_{\alpha }\right)}$ or ${\mathrm{PF}}^{\left(f_{\alpha },f_{\alpha }\right)}$. Since $D_{f_{2}}\left(P∥Q\right)\leq {\left(1+\left(\alpha -1\right)D_{f_{\alpha }}\left(P∥Q\right)\right)}^{1/(\alpha -1)}-1$ (see [106] (Corollary 5.6)), one can justify $I_{f_{\alpha }}$ as a measure of privacy and utility in a similar way as $I_{f_{2}}$.

We end this section by a remark about estimating the measures studied in this section. While we consider information-theoretic regime where the underlying distribution $P_{XY}$ is known, in practice only samples $\left(x_{i},y_{i}\right)$ are given. Consequently, the de facto guarantees of bottleneck problems might be considerably different from those shown in this work. It is therefore essential to asses the guarantees of bottleneck problems when accessing only samples. To do so, one must derive bounds on the discrepancy between $P_{c}$, $I_{\alpha }$, and $I_{f}$ computed on the empirical distribution and the true (unknown) distribution. These bounds can then be used to shed light on the de facto guarantee of the bottleneck problems. Relying on [34] (Theorem 1), one can obtain that the gaps between the measures $P_{c}$, $I_{\alpha }$, and $I_{f}$ computed on empirical distributions and the true one scale as $O(1/\sqrt{n})$ where *n* is the number of samples. This is in contrast with mutual information for which the similar upper bound scales as $O(\log n/\sqrt{n})$ as shown in [33]. Therefore, the above measures appear to be easier to estimate than mutual information.

## 4. Summary and Concluding Remarks

Following the recent surge in the use of information bottleneck (IB) and privacy funnel (PF) in developing and analyzing machine learning models, we investigated the functional properties of these two optimization problems. Specifically, we showed that IB and PF correspond to the upper and lower boundary of a two-dimensional convex set M={(I(X;T),I(Y;T)): *Y* 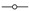 *X* 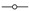 *T*} where $\left(X,Y\right)∼P_{XY}$ represents the observable data *X* and target feature *Y* and the auxiliary random variable *T* varies over all possible choices satisfying the Markov relation *Y* 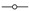 *X* 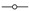 *T*. This unifying perspective on IB and PF allowed us to adapt the classical technique of Witsenhausen and Wyner [3] devised for computing IB to be applicable for PF as well. We illustrated this by deriving a closed form expression for PF in the binary case—a result reminiscent of the Mrs. Gerber’s Lemma [2] in information theory literature. We then showed that both IB and PF are closely related to several information-theoretic coding problems such as noisy random coding, hypothesis testing against independence, and dependence dilution. While these connections were partially known in previous work (see e.g., [29,30]), we show that they lead to an improvement on the cardinality of *T* for computing IB. We then turned our attention to the continuous setting where *X* and *Y* are continuous random variables. Solving the optimization problems in IB and PF in this case without any further assumptions seems a difficult challenge in general and leads to theoretical results only when (X,Y) is jointly Gaussian. Invoking recent results on the entropy power inequality [25] and strong data processing inequality [27], we obtained tight bounds on IB in two different cases: (1) when *Y* is a Gaussian perturbation of *X* and (2) when *X* is a Gaussian perturbation of *Y*. We also utilized the celebrated I-MMSE relationship [107] to derive a second-order approximation of PF when *T* is considered to be a Gaussian perturbation of *X*.

In the second part of the paper, we argue that the choice of (Shannon’s) mutual information in both IB and PF does not seem to carry specific operational significance. It does, however, have a desirable practical consequence: it leads to self-consistent equations [1] that can be solved iteratively (without any guarantee to convergence though). In fact, this property is unique to mutual information among other existing information measures [99]. Nevertheless, we argued that other information measures might lead to better interpretable guarantee for both IB and PF. For instance, statistical accuracy in IB and privacy leakage in PF can be shown to be precisely characterized by probability of correctly guessing (aka Bayes risk) or minimum mean-squared error (MMSE). Following this observation, we introduced a large family of optimization problems, which we call bottleneck problems, by replacing mutual information in IB and PF with Arimoto’s mutual information [22] or *f*-information [23]. Invoking results from [33,34], we also demonstrated that these information measures are in general easier to estimate from data than mutual information. Similar to IB and PF, the bottleneck problems were shown to be fully characterized by boundaries of a two-dimensional convex set parameterized by two real-valued non-negative functions Φ and Ψ. This perspective enabled us to generalize the technique used to compute IB and PF for evaluating bottleneck problems. Applying this technique to the binary case, we derived closed form expressions for several bottleneck problems.

## Appendix A. Proofs from Section 2

**Proof** **of Theorem 1.**

- Note that R=0 in optimization problem (4) implies that *X* and *T* are independent. Since Y,X and *T* form Markov chain *Y*  *Y*  *T*, independent of *X* and *T* implies independence of *Y* and *T* and thus I(Y;T)=0. Similarly for PF(0).
- Since I(X;T)≤H(X) for any random variable *T*, we have T=X satisfies the information constraint I(X;T)≤R for R≥H(X). Since I(Y;T)≤I(Y;X), this choice is optimal. Similarly for PF, the constraint I(X;T)≥r for r≥H(X) implies T=X. Hence, PF(r)=I(Y;X).
- The upper bound on IB follows from the data processing inequality: I(Y;T)≤min{I(X;T),I(X;Y)} for all *T* satisfying the Markov condition *Y*  *X*  *T*.
- To prove the lower bound on PF, note that
  I(Y;T)=I(X;T)−I(X;T|Y)≥I(X;T)−H(X|Y).
- The concavity of R↦IB(R) follows from the fact it is the upper boundary of the convex set M, defined in (6). This in turn implies the continuity of IB(·). Monotonicity of R↦IB(R) follows from the definition. Strict monotonicity follows from the convexity and the fact that IB(H(X))=I(X;Y).
- Similar as above.
- The differentiability of the map R↦IB(R) follows from [94] (Lemma 6). This result in fact implies the differentiability of the map r↦PF(r) as well. Continuity of the derivative of IB and PF on (0,H(X)) is a straightforward application of [108] (Theorem 25.5).
- Monotonicity of mappings $R\mapsto \frac{\mathrm{IB}(R)}{R}$ and $r\mapsto \frac{\mathrm{PF}(r)}{r}$ follows from the concavity and convexity of IB(·) and PF(·), respectively.
- Strict monotonicity of IB(·) and PF(·) imply that the optimization problems in (4) and (5) occur when the inequality in the constraints becomes equality. □

**Proof** **of Theorem 3.** Recall that, according to Theorem 1, the mappings R↦IB(R) and r↦PF(r) are concave and convex, respectively. This implies that IB(R) (resp. PF(r)) lies above (resp. below) the chord connecting (0,0) and (H(X,I(X;Y)). This proves the lower bound (resp. upper bound) $\mathrm{IB}\left(R\right)\geq R\frac{I(X;Y)}{H(X)}$ (resp. $\mathrm{PF}\left(r\right)\leq r\frac{I(X;Y)}{H(X)}$). In light of the convexity of PF and monotonicity of $r\mapsto \frac{\mathrm{PF}(r)}{r}$, we can write

where the last equality is due to [13] (Lemma 4) and $Q_{Y}$ is the output distribution of the channel $P_{Y|X}$ when the input is distributed according to $Q_{X}$. Similarly, we can write

where the last equality is due to [82] (Theorem 4). □

**Proof** **of Theorem 5.** Let $T_{n}$ be an optimal summeries of $X^{n}$, that is, it satisfies *T_n_*  *X^n^*  *Y^n^* and $I(X^{n};T_{n})=nR$. We can write

$$I\left(X^{n},T_{n}\right)=H\left(X^{n}\right)-H\left(X^{n}|T_{n}\right)=\sum_{k=1}^{n}\left[H\left(X_{k}\right)-H\left(X_{k}|X^{k-1},T_{n}\right)\right]=\sum_{k=1}^{n}I\left(X_{k};X^{k-1},T_{n}\right),$$

and hence, if $R_{k}≔I\left(X_{k};X^{k-1},T_{n}\right)$, then we have

(A1) $$R=\frac{1}{n}\sum_{k=1}^{n}R_{k}.$$

We can similarly write

$$\begin{array}{ccc} I(Y^{n},T_{n}) & = & H\left(Y^{n}\right)-H\left(Y^{n}|T_{n}\right)=\sum_{k=1}^{n}\left[H\left(Y_{k}\right)-H\left(Y_{k}|Y^{k-1},T_{n}\right)\right]\\ & \leq & \sum_{k=1}^{n}\left[H\left(Y_{k}\right)-H\left(Y_{k}|Y^{k-1},X^{k-1},T_{n}\right)\right]\\ & = & \sum_{k=1}^{n}\left[H\left(Y_{k}\right)-H\left(Y_{k}|X^{k-1},T_{n}\right)\right]=\sum_{k=1}^{n}I\left(Y_{k};X^{k-1},T_{n}\right). \end{array}$$

Since we have $\left(T_{n},X^{k-1}\right)$  *X_k_*  *Y_k_* for every k∈[n], we conclude from the above inequality that

(A2) $$I\left(Y^{n},T_{n}\right)\leq \sum_{k=1}^{n}I\left(Y_{k};X^{k-1},T_{n}\right)\leq \sum_{k=1}^{n}\mathrm{IB}\left(P_{XY},R_{k}\right)\leq n\mathrm{IB}\left(P_{XY},R\right),$$

where the last inequality follows from concavity of the map $x\mapsto \mathrm{IB}(P_{XY},x)$ and (A1). Consequently, we obtain

(A3) $$\mathrm{IB}\left(P_{X^{n}Y^{n}},nR\right)\leq n\mathrm{IB}\left(P_{XY},R\right).$$

To prove the other direction, let $P_{T|X}$ be an optimal channel in the definition of IB, i.e., I(X;T)=R and $\mathrm{IB}\left(P_{XY},R\right)=I\left(Y;T\right)$. Then using this channel *n* times for each pair $\left(X_{i},Y_{i}\right)$, we obtain $T^{n}=\left(T_{1},\ldots ,T_{n}\right)$ satisfying *T^n^*  *X^n^*  *Y^n^*. Since $I\left(X^{n};T^{n}\right)=nI\left(X;T\right)=nR$ and $I\left(Y^{n};T^{n}\right)=nI\left(Y;T\right)$, we have $\mathrm{IB}\left(P_{X^{n}Y^{n}},nR\right)\geq n\mathrm{IB}\left(P_{XY},R\right).$ This, together with (A3), concludes the proof. □

**Proof** **of Theorem 4.** First notice that

where the last equality is due to [82] (Theorem 4). Similarly,

where the last equality is due to [13] (Lemma 4). Fix $x_{0}\in X$ with $P_{X}\left(x_{0}\right)>0$ and let *T* be a Bernoulli random variable specified by the following channel

$$P_{T|X}\left(1|x\right)=\delta 1_{\left\{x=x_{0}\right\}},$$

for some δ>0. This channel induces $T∼\mathrm{Bernoulli}(\delta P_{X}\left(x_{0}\right))$, $P_{Y|T}\left(y|1\right)=P_{Y|X}\left(y|x_{0}\right)$, and

$$P_{Y|T}\left(y|0\right)=\frac{P_{Y}\left(y\right)-P_{XY}\left(x_{0},y\right)}{1-\delta P_{X}\left(x_{0}\right)}.$$

It can be verified that

$$I\left(X;T\right)=-\delta P_{X}\left(x_{0}\right)\log P_{X}\left(x_{0}\right)+o\left(\delta \right),$$

and

$$I\left(Y;T\right)=\delta P_{X}\left(x_{0}\right)D_{\mathrm{KL}}\left(P_{Y|X}\left(\cdot |x_{0}\right)∥P_{Y}\left(\cdot \right)\right)+o\left(\delta \right).$$

Setting

$$\delta =\frac{r}{-P_{X}\left(x_{0}\right)\log P_{X}\left(x_{0}\right)},$$

we obtain

$$I\left(Y;T\right)=\frac{D_{\mathrm{KL}}\left(P_{Y|X}\left(\cdot |x_{0}\right)∥P_{Y}\left(\cdot \right)\right)}{-\log P_{X}\left(x_{0}\right)}r+o\left(r\right),$$

and hence

$$\mathrm{PF}\left(r\right)\leq \frac{D_{\mathrm{KL}}\left(P_{Y|X}\left(\cdot |x_{0}\right)∥P_{Y}\left(\cdot \right)\right)}{-\log P_{X}\left(x_{0}\right)}r+o\left(r\right).$$

Since $x_{0}$ is arbitrary, the result follows. The proof for IB follows similarly. □

**Proof** **of Lemma 1.** When *Y* is an erasure of *X*, i.e., Y=X∪{⊥} with $P_{Y|X}\left(x|x\right)=1-\delta$ and $P_{Y|X}\left(⊥|x\right)=\delta$, it is straightforward to verify that $D_{\mathrm{KL}}\left(Q_{Y}∥P_{Y}\right)=\left(1-\delta \right)D_{\mathrm{KL}}\left(Q_{X}∥P_{X}\right)$ for every $P_{X}$ and $Q_{X}$ in P(X). Consequently, we have

$$\underset{Q_{X}\neq P_{X}}{\inf}\frac{D_{\mathrm{KL}}\left(Q_{Y}∥P_{Y}\right)}{D_{\mathrm{KL}}\left(Q_{X}∥P_{X}\right)}=\underset{Q_{X}\neq P_{X}}{\sup}\frac{D_{\mathrm{KL}}\left(Q_{Y}∥P_{Y}\right)}{D_{\mathrm{KL}}\left(Q_{X}∥P_{X}\right)}=1-\delta .$$

Hence, Theorem 3 gives the desired result. To prove the second part, i.e., when *X* is an erasure of *Y*, we need an improved upper bound of PF. Notice that if perfect privacy occurs for a given $P_{XY}$, then the upper bound for PF(r) in Theorem 3 can be improved:

(A4) $$\mathrm{PF}\left(r\right)\leq \left(r-r_{0}\right)\frac{I(X;Y)}{H\left(X\right)-r_{0}},$$

where $r_{0}$ is the largest r≥0 such that PF(r)=0. Here, we show that $r_{0}=H\left(X|Y\right)$. This suffices to prove the result as (A4), together with Theorem 1, we have

$$\max\left\{r-H\left(X|Y\right),0\right\}\leq \mathrm{PF}\leq \left(r-r_{0}\right)\frac{I(X;Y)}{H\left(X\right)-r_{0}}=\left(r-H\left(X|Y\right)\right).$$

To show that PF(H(X|Y))=0, consider the channel $P_{T|X}\left(t|x\right)=\frac{1}{\left|Y\right|}1_{\left\{t\neq ⊥,x\neq ⊥\right\}}$ and $P_{T|X}\left(⊥|⊥\right)=1.$ It can be verified that this channel induces *T* which is independent of *Y* and that

$$I\left(X;T\right)=H\left(T\right)-H\left(T|X\right)=H\left(\frac{1-\delta }{\left|Y\right|},\cdots ,\frac{1-\delta }{\left|Y\right|},\delta \right)-\left(1-\delta \right)\log\left|Y\right|=h_{b}\left(\delta \right)=H\left(X|Y\right),$$

where $h_{b}\left(\delta \right)≔-\delta \log\delta -\left(1-\delta \right)\log\left(1-\delta \right)$ is the binary entropy function. □

**Proof** **of Lemma 4.** As mentioned earlier, Equation (24) was proved in [2]. We thus give a proof only for (25). Consider the problem of minimizing the Lagrangian $L_{\mathrm{PF}}\left(\beta \right)$ (20) for $\beta \geq \beta_{\mathrm{PF}}$. Let $X^{\prime }∼Q_{X}=\mathrm{Bernoulli}\left(q\right)$ for some q∈(0,1) and $Y^{\prime }$ be the result of passing $X^{\prime }$ through BSC(δ), i.e., $Y^{\prime }∼\mathrm{Bernoulli}\left(q*\delta \right)$. Recall that $F_{\beta }\left(q\right)≔F_{\beta }\left(Q_{X}\right)=h_{b}\left(q*\delta \right)-\beta h_{b}\left(q\right)$. It suffices to compute $K_{\cap }\left[F_{\beta }\left(q\right)\right]$ the upper concave envelope of $q\mapsto F_{\beta }\left(q\right)$. It can be verified that $\beta_{\mathrm{IB}}\leq {\left(1-2\delta \right)}^{2}$ and hence for all $\beta \geq {\left(1-2\delta \right)}^{2}$, $K_{\cap }\left[F_{\beta }\left(q\right)\right]=F_{\beta }\left(0\right)$. A straightforward computation shows that $F_{\beta }\left(q\right)$ is symmetric around $q=\frac{1}{2}$ and is also concave in a region around $q=\frac{1}{2}$, where it reaches its local maximum. Hence, if β is such that

- $F_{\beta }\left(\frac{1}{2}\right)<F_{\beta }\left(0\right)$ (see Figure 4a), then $K_{\cap }\left[F_{\beta }\left(q\right)\right]$ is given by the convex combination of $F_{\beta }\left(0\right)$ and $F_{\beta }\left(1\right)$.
- $F_{\beta }\left(\frac{1}{2}\right)=F_{\beta }\left(0\right)$ (see Figure 4b), then $K_{\cap }\left[F_{\beta }\left(q\right)\right]$ is given by the convex combination of $F_{\beta }\left(0\right)$ and $F_{\beta }\left(\frac{1}{2}\right)$ and $F_{\beta }\left(1\right)$.
- $F_{\beta }\left(\frac{1}{2}\right)>F_{\beta }\left(0\right)$ (see Figure 4c), then there exists $q_{\beta }\in \left[0,\frac{1}{2}\right]$ such that for $q\leq q_{\beta }$, $K_{\cap }\left[F_{\beta }\left(q\right)\right]$ is given by the convex combination of $F_{\beta }\left(0\right)$ and $F_{\beta }\left(q_{\beta }\right)$.

Hence, assuming $p\leq \frac{1}{2}$, we can construct $T^{*}$ that maximizes H(Y|T)−βH(X|T) in three different cases corresponding three cases above:

- In the first case, $T^{*}$ is binary and we have $P_{X|T^{*}=0}=\mathrm{Bernoulli}\left(0\right)$ and $P_{X|T^{*}=1}=\mathrm{Bernoulli}\left(1\right)$ with $P_{T^{*}}=\mathrm{Bernoulli}\left(p\right)$.
- In the second case, $T^{*}$ is ternary and we have $P_{X|T^{*}=0}=\mathrm{Bernoulli}\left(0\right)$, $P_{X|T^{*}=1}=\mathrm{Bernoulli}\left(1\right)$, and $P_{X|T^{*}=2}=\mathrm{Bernoulli}\left(\frac{1}{2}\right)$ with $P_{T^{*}}=\left(1-p-\frac{\alpha }{2},p-\frac{\alpha }{2},\alpha \right)$ for some α∈[0,2p].
- In the third case, $T^{*}$ is again binary and we have $P_{X|T^{*}=0}=\mathrm{Bernoulli}\left(0\right)$ and $P_{X|T^{*}=1}=\mathrm{Bernoulli}\left(\frac{p}{\alpha }\right)$ with $P_{T^{*}}=\mathrm{Bernoulli}\left(\alpha \right)$ for some α∈[2p,1].

Combining these three cases, we obtain the result in (25).□

**Proof** **of Lemma 2.** Let $X=Y+\sigma N^{G}$ where σ>0 and $N^{G}∼N\left(0,1\right)$ is independent of *Y*. According to the improved entropy power inequality proved in [25] (Theorem 1), we can write

$$e^{2(H(X)-I(Y;T))}\geq e^{2(H(Y)-I(X;T))}+2\pi e\sigma^{2},$$

for any random variable *T* forming *Y*  *X*  *T*. This, together with Theorem 5, implies the result. □

**Proof** **of Corollary 1.** Since (X,Y) are jointly Gaussian, we can write $X=Y+\sigma N^{G}$ where $\sigma =\sigma_{Y}\frac{\sqrt{1-\rho^{2}}}{\rho }$ and $\sigma_{Y}^{2}$ is the variance of *Y*. Applying Lemma 2 and noticing that $H\left(X\right)=\frac{1}{2}\log\left(2\pi e\left(\sigma_{Y}^{2}+\sigma^{2}\right)\right)$, we obtain

(A5) $$I\left(Y;T\right)\leq \frac{1}{2}\log\frac{\sigma^{2}+\sigma_{Y}^{2}}{\sigma^{2}+\sigma_{Y}^{2}e^{-2I(X;T)}}=\frac{1}{1-\rho^{2}+\rho^{2}e^{-2I(X;T)}},$$

for all channels $P_{T|X}$ satisfying *Y*  *X*  *T*. This bound is attained by Gaussian $P_{T|X}$. Specifically, assuming $T+X+\tilde{\sigma }M^{G}$ where ${\tilde{\sigma }}^{2}=\sigma_{Y}^{2}\frac{e^{-2R}}{\rho^{2}\left(1-e^{-2R}\right)}$ for R≥0 and $M^{G}∼N\left(0,{\tilde{\sigma }}^{2}\right)$ independent of *X*, it can be easily verified that I(X;T)=R and $I\left(Y;T\right)=\frac{1}{1-\rho^{2}+\rho^{2}e^{-2R}}$. This, together with (A5), implies $\mathrm{IB}\left(R\right)=\frac{1}{1-\rho^{2}+\rho^{2}e^{-2R}}.$ □

Next, we wish to prove Theorem 6. However, we need the following preliminary lemma before we delve into its proof.

**Lemma** **A1.**
*Let X and Y be continuous correlated random variables with $E[X^{2}]<\infty$ and $E[Y^{2}]<\infty$. Then the mappings $\sigma \mapsto I(X;T_{\sigma })$ and $\sigma \mapsto I(Y;T_{\sigma })$ are continuous, strictly decreasing, and*

$$I\left(X;T_{\sigma }\right)\to 0,\;\mathrm{and}\;I\left(Y;T_{\sigma }\right)\to 0\;\mathrm{as}\;\sigma \to \infty .$$

**Proof.** The finiteness of $E[X^{2}]$ and $E[Y^{2}]$ imply that H(X) and H(Y) are finite. A straightforward application of the entropy power inequality (cf. [109] (Theorem 17.7.3)) implies that $H(T_{\sigma })$ is also finite. Thus, $I(X;T_{\sigma })$ and $I(Y;T_{\sigma })$ are well-defined. According to the data processing inequality, we have $I\left(X;T_{\sigma +\delta }\right)<I\left(X;T_{\sigma }\right)$ for all δ>0 and also $I\left(Y;T_{\sigma +\delta }\right)\leq I\left(Y;T_{\sigma }\right)$ where the equality occurs if and only if *X* and *Y* are independent. Since, bu assumption *X* and *Y* correlated, it follows $I\left(Y;T_{\sigma +\delta }\right)<I\left(Y;T_{\sigma }\right)$. Thus, both $I(X;T_{\sigma })$ and $I(Y;T_{\sigma })$ are strictly decreasing. For the proof of continuity, we consider two cases σ=0 and σ>0 separately. We first give the poof for $I(X;T_{\sigma })$. Since $H\left(\sigma N^{G}\right)=\frac{1}{2}\log\left(2\pi e\sigma^{2}\right)$, we have $\lim_{\sigma \to 0}H\left(\sigma N^{G}\right)=\infty$ and thus $\lim_{\sigma \to 0}I\left(X;T_{\sigma }\right)=\infty$ that is equal to $I(X;T_{0})$. For σ>0, let $\sigma_{n}$ be a sequence of positive numbers converging to σ. In light of de Bruijn’s identity (cf. [109] (Theorem 17.7.2)), we have $H\left(T_{\sigma_{n}}\right)\to H\left(T_{\sigma }\right)$, implying the continuity of $\sigma \mapsto I(X;T_{\sigma })$. Next, we prove the continuity of $\sigma \mapsto I(Y;T_{\sigma })$. For the sequence of positive numbers $\sigma_{n}$ converging to σ>0, we have $I\left(Y;T_{\sigma_{n}}\right)=H\left(T_{\sigma_{n}}\right)-H\left(T_{\sigma_{n}}|Y\right)$. We only need to show $H\left(T_{\sigma_{n}}|Y\right)\to H\left(T_{\sigma }|Y\right)$. Invoking again de Brujin’s identity, we obtain $H\left(T_{\sigma_{n}}|Y=y\right)\to H\left(T_{\sigma }|Y=y\right)$ for each y∈Y. The desired result follows from dominated convergence theorem. Finally, the The continuity of $\sigma \mapsto I(Y;T_{\sigma })$ when σ=0 follows from [110] (p. 2028) stating that $H\left(T_{\sigma_{n}}|Y=y\right)\to H\left(X|Y=y\right)$ and then applying dominated convergence theorem. Note that

$$0\leq I\left(Y;T_{\sigma }\right)\leq I\left(X;T_{\sigma }\right)\leq \frac{1}{2}\log\left(1+\frac{\sigma_{X}^{2}}{\sigma^{2}}\right),$$

where $\sigma_{X}^{2}$ is the variance of *X* and the last inequality follows from the fact that $I(X;X+\sigma N^{G})$ is maximized when *X* is Gaussian. Since by assumption $\sigma_{X}<\infty$, it follows that both $I(X;T_{\sigma })$ and $I(Y;T_{\sigma })$ converge to zero as σ→∞. □

In light of this lemma, there exists a unique σ≥0 such that $I(X;T_{\sigma })=r$. Let $\sigma_{r}$ denote such σ. Therefore, we have $\mathrm{PF}\left(r\right)=I\left(Y;T_{\sigma_{r}}\right).$ This enables us to prove Theorem 6.

**Proof** **of Theorem 6.** The proof relies on the I-MMSE relation in information theory literature. We briefly describe it here for convenience. Given any pair of random variables *U* and *V*, the minimum mean-squared error (MMSE) of estimating *U* given *V* is given by

$$\mathrm{mmse}\left(U|V\right)≔\underset{f}{\inf}E\left[{\left(U-f\left(V\right)\right)}^{2}\right]=E\left[{\left(U-E[U|V]\right)}^{2}\right]=E\left[\mathrm{var}\left(U|V\right)\right],$$

where the infimum is taken over all measurable functions *f* and $\mathrm{var}\left(U|V\right)=E[{\left(U-E\left[U|V\right]\right)}^{2}|V]$. Guo et al. [107] proved the following identity, which is referred to as I-MMSE formula, relating the input-output mutual information of the additive Gaussian channel $T_{\sigma }=X+\sigma N^{G}$, where $N^{G}∼N\left(0,1\right)$ is independent of *X*, with the MMSE of the input given the output:

(A6) $$\frac{d}{d(\sigma^{2})}I\left(X;T_{\sigma }\right)=-\frac{1}{2\sigma^{4}}\mathrm{mmse}\left(X|T_{\sigma }\right).$$

Since *Y*, *X*, and $T_{\sigma }$ form the Markov chain *Y*  *X*  *T*_*σ*_, it follows that $I\left(Y;T_{\sigma }\right)=I\left(X;T_{\sigma }\right)-I\left(X;T_{\sigma }|Y\right)$. Thus, two applications of (A6) yields

(A7) $$\frac{d}{d(\sigma^{2})}I\left(Y;T_{\sigma }\right)=-\frac{1}{2\sigma^{4}}\left[\mathrm{mmse}\left(X|T_{\sigma }\right)-\mathrm{mmse}\left(X|T_{\sigma },Y\right)\right].$$

The second derivative of $I(X;T_{\sigma })$ and $I(Y;T_{\sigma })$ are also known via the formula [111] (Proposition 9)

(A8) $$\frac{d}{d(\sigma^{2})}\mathrm{mmse}\left(X|T_{\sigma }\right)=\frac{1}{\sigma^{4}}E\left[{\mathrm{var}}^{2}\left(X|T_{\sigma }\right)\right]\;\mathrm{and}\;\frac{d}{d(\sigma^{2})}\mathrm{mmse}\left(X|T_{\sigma },Y\right)=\frac{1}{\sigma^{4}}E\left[{\mathrm{var}}^{2}\left(X|T_{\sigma },Y\right)\right].$$

With these results in mind, we now begin the proof. Recall that $\sigma_{r}$ is the unique σ such that $I(X;T_{\sigma })=r$, thus implying ${\mathrm{PF}}^{G}\left(r\right)=I\left(Y;T_{\sigma_{r}}\right)$. We have

(A9) $$\frac{d}{dr}{\mathrm{PF}}^{G}\left(r\right)={\left[\frac{d}{d(\sigma^{2})}I\left(Y;T_{\sigma }\right)\right]}_{\sigma =\sigma_{r}}\frac{d}{dr}\sigma_{r}^{2}.$$

To compute the derivative of PF(r), we therefore need to compute the derivative of $\sigma_{r}^{2}$ with respect to *r*. To do so, notice that from the identity $I(X;T_{\sigma_{r}})=r$ we can obtain

$$1=\frac{d}{dr}I\left(X;T_{\sigma_{r}}\right)={\left[\frac{d}{d(\sigma^{2})}I\left(X;T_{\sigma_{r}}\right)\right]}_{\sigma =\sigma_{r}}\frac{d}{dr}\sigma_{r}^{2}=-\frac{1}{2\sigma^{4}}\mathrm{mmse}\left(X|T_{\sigma_{r}}\right)\frac{d}{dr}\sigma_{r}^{2},$$

implying

$$\frac{d}{dr}\sigma_{r}^{2}=\frac{-2\sigma^{4}}{\mathrm{mmse}(X|T_{\sigma_{r}})}.$$

Plugging this identity into (A9) and invoking (A7), we obtain

(A10) $$\frac{d}{dr}{\mathrm{PF}}^{G}\left(r\right)=\frac{\mathrm{mmse}\left(X|T_{\sigma_{r}}\right)-\mathrm{mmse}\left(X|T_{\sigma_{r}},Y\right)}{\mathrm{mmse}(X|T_{\sigma_{r}})}.$$

The second derivative can be obtained via (A8)

$$\frac{d^{2}}{dr^{2}}{\mathrm{PF}}^{G}\left(r\right)=2\frac{E[{\mathrm{var}}^{2}\left(X|T_{\sigma_{r}},Y\right)]}{{\mathrm{mmse}}^{2}\left(X|T_{\sigma_{r}}\right)}-2E\left[{\mathrm{var}}^{2}\left(X|T_{\sigma_{r}}\right)\right]\frac{\mathrm{mmse}(X|T_{\sigma_{r}},Y)}{{\mathrm{mmse}}^{3}\left(X|T_{\sigma_{r}}\right)}.$$

Since $\sigma_{r}\to \infty$ as r→0, we can write

$$\frac{d}{dr}{\mathrm{PF}}^{G}\left(r\right){|}_{r=0}=\frac{\sigma_{X}^{2}-E\left[\mathrm{var}\left(X|Y\right)\right]}{\sigma_{X}}=\frac{\mathrm{var}(E[X|Y])}{\sigma_{X}^{2}}=\eta \left(X,Y\right),$$

where var(E[X|Y]) is the variance of the conditional expectation *X* given *Y* and the last equality comes from the law of total variance. and

$$\frac{d^{2}}{dr^{2}}{\mathrm{PF}}^{G}\left(r\right){|}_{r=0}=\frac{2}{\sigma_{X}^{4}}\left[E\left[{\mathrm{var}}^{2}\left(X|Y\right)\right]-\sigma_{X}^{2}E\left[\mathrm{var}\left(X|Y\right)\right]\right].$$

Taylor expansion of PF(r) around r=0 gives the result. □

**Proof** **of Theorem 9.** The main ingredient of this proof is a result by Jana [112] (Lemma 2.2) which provides a tight cardinality bound for the auxiliary random variables in the canonical problems in network information theory (including noisy source coding problem described in Section 2.3). Consider a pair of random variables $\left(X,Y\right)∼P_{XY}$ and let $d:Y\times \hat{Y}\to R$ be an arbitrary distortion measure defined for arbitrary reconstruction alphabet $\hat{Y}$. □ **Theorem** **A1** ([112])**.** *Let A be the set of all pairs (R,D) satisfying*

I(X;T)≤RandE[d(Y,ψ(T))]≤D,

*for some mapping $\psi :T\to \hat{Y}$ and some joint distributions $P_{XYT}=P_{XY}P_{T|X}$. Then every extreme points of A corresponds to some choice of auxiliary variable T with alphabet size |T|≤|X|.*
Measuring the distortion in the above theorem in terms of the logarithmic loss as in (27), we obtain that

$$A=\{\left(R,D\right)\in R_{+}^{2}:\;R\geq R^{\mathrm{noisy}}\left(D\right)\},$$

where $R^{\mathrm{noisy}}\left(D\right)$ is given in (29). We observed in Section 2.3 that IB is fully characterized by the mapping $D\mapsto R^{\mathrm{noisy}}\left(D\right)$ and thus by A. In light of Theorem A1, all extreme points of A are achieved by a choice of *T* with cardinality size |T|≤|X|. Let $\left\{\left(R_{i},D_{i}\right)\right\}$ be the set of extreme points of A each constructed by channel $P_{T_{i}|X}$ and mapping $\psi_{i}$. Due to the convexity of A, each point (R,D)∈A is expressed as a convex combination of $\left\{\left(R_{i},D_{i}\right)\right\}$ with coefficient $\left\{\lambda_{i}\right\}$; that is there exists a channel $P_{T|X}=\sum_{i}\lambda_{i}P_{T_{i}|X}$ and a mapping $\psi \left(T\right)=\sum_{i}\lambda_{i}\psi_{i}\left(T_{i}\right)$ such that I(X;T)=R and E[d(Y,ψ(T))]=D. This construction, often termed timesharing in information theory literature, implies that all points in A (including the boundary points) can be achieved with a variable *T* with |T|≤|X|. Since the boundary of A is specified by the mapping R↦IB(R), we conclude that IB(R) is achieved by a variable *T* with cardinality |T|≤|X| for very R<H(X).

**Proof** **of Lemma 5.** The following proof is inspired by [32] (Proposition 1). Let X={1,…,m}. We sort the elements in X such that

$$P_{X}\left(1\right)D_{\mathrm{KL}}\left(P_{Y|X=1}∥P_{Y}\right)\geq \cdots \geq P_{X}\left(m\right)D_{\mathrm{KL}}\left(P_{Y|X=m}∥P_{Y}\right).$$

Now consider the function f:X→[M] given by f(x)=x if x<M and f(x)=M if x≥M where $M=e^{R}$. Let Z=f(X). We have $P_{Z}\left(i\right)=P_{X}\left(i\right)$ if i<M and $P_{Z}\left(M\right)=\sum_{j\geq M}P_{X}\left(j\right)$. We can now write

$$\begin{array}{cc} I(Y;Z) & =\sum_{i=1}^{M-1}P_{X}\left(i\right)D\left(P_{Y|X=i}∥P_{Y}\right)+P_{Z}\left(M\right)D\left(P_{Y|Z=M}∥P_{Y}\right)\\ \; & \geq \sum_{i=1}^{M-1}P_{X}\left(i\right)D\left(P_{Y|X=i}∥P_{Y}\right)\\ \; & \geq \frac{M-1}{\left|X\right|}\sum_{i\in X}P_{X}\left(i\right)D\left(P_{Y|X=i}∥P_{Y}\right)\\ \; & =\frac{M-1}{\left|X\right|}I\left(X;Y\right). \end{array}$$

Since f(X) takes values in [M], it follows that H(f(X))≤R. Consequently, we have

$$\mathrm{dIB}\left(P_{XY},R\right)\geq \underset{f:X\to [M]}{\sup}I\left(Y;f\left(X\right)\right)\geq \frac{M-1}{\left|X\right|}I\left(X;Y\right).$$

For the privacy funnel, the proof proceeds as follows. We sort the elements in X such that

$$P_{X}\left(1\right)D_{\mathrm{KL}}\left(P_{Y|X=1}∥P_{Y}\right)\leq \cdots \leq P_{X}\left(m\right)D_{\mathrm{KL}}\left(P_{Y|X=m}∥P_{Y}\right).$$

Consider now the function f:X→[M] given by f(x)=x if x<M and f(x)=M if x≥M. As before, let Z=f(X). Then, we can write,

$$\begin{array}{cc} I(Y;Z) & =\sum_{i=1}^{M-1}P_{X}\left(i\right)D\left(P_{Y|X=i}∥P_{Y}\right)+P_{Z}\left(M\right)D\left(P_{Y|Z=M}∥P_{Y}\right)\\ \; & \leq \frac{M-1}{\left|X\right|}\sum_{i\in X}P_{X}\left(i\right)D\left(P_{Y|X=i}∥P_{Y}\right)+P_{Z}\left(M\right)D\left(P_{Y|Z=M}∥P_{Y}\right)\\ \; & =\frac{M-1}{\left|X\right|}I\left(X;Y\right)+P_{Z}\left(M\right)\sum_{y\in Y}P_{Y|Z}\left(y|M\right)\log\frac{P_{Y|Z}\left(y|M\right)}{P_{Y}\left(y\right)}\\ \; & \leq \frac{M-1}{\left|X\right|}I\left(X;Y\right)+\mathrm{Pr}\left(X\geq M\right)\sum_{y\in Y}\left[\sum_{i}P_{Y|X}\left(y|i\right)\frac{P_{X}\left(i\right)1_{\left\{i\geq M\right\}}}{\mathrm{Pr}(X\geq M)}\right]\log\frac{\sum_{i}P_{Y|X}\left(y|i\right)\frac{P_{X}\left(i\right)1_{\left\{i\geq M\right\}}}{\mathrm{Pr}(X\geq M)}}{\sum_{i}P_{Y|X}\left(y|i\right)P_{X}\left(i\right)}\\ \; & \leq \frac{M-1}{\left|X\right|}I\left(X;Y\right)+\sum_{y\in Y}\sum_{i\geq M}P_{Y|X}\left(y|i\right)P_{X}\left(i\right)\log\frac{1}{\mathrm{Pr}(X\geq M)}\\ \; & =\frac{M-1}{\left|X\right|}I\left(X;Y\right)+\mathrm{Pr}\left(X\geq M\right)\log\frac{1}{\mathrm{Pr}(X\geq M)} \end{array}$$

where the last inequality is due to the log-sum inequality. □

**Proof** **of Lemma 6.** Employing the same argument as in the proof of [32] (Theorem 3), we obtain that there exists a function f:X→[M] such that

(A11) I(Y;f(X))≥ηI(X;Y)

for any η∈(0,1) and

$$M\leq 4+\frac{4}{(1-\eta )\log2}\log\frac{2\alpha }{\left(1-\eta )I(X;Y\right)}.$$

Since $h_{b}^{-1}\left(x\right)\leq \frac{x\log2}{\log\frac{1}{x}}$ for all x∈(0,1], it follows from above that (noticing that I(X;Y)≤α)

$$M\leq 4+\frac{I(X;Y)}{2\alpha }\frac{1}{h_{b}^{-1}\left(\zeta \right)},$$

where $\zeta ≔\frac{\left(1-\eta )I(X;Y\right)}{2\alpha }$. Rearranging this, we obtain

$$h_{b}^{-1}\left(\zeta \right)\leq \frac{I(X;Y)}{2\alpha (M-4)}.$$

Assuming M≥5, we have $\frac{I(X;Y)}{2\alpha (M-4)}\leq \frac{1}{2}$ and hence

$$\zeta \leq h_{b}\left(\frac{I(X;Y)}{2\alpha (M-4)}\right),$$

implying

$$\eta \geq 1-\frac{2\alpha }{I(X;Y)}h_{b}\left(\frac{I(X;Y)}{2\alpha (M-4)}\right).$$

Plugging this into (A11), we obtain

$$I\left(Y;f\left(X\right)\right)\geq I\left(X;Y\right)-2\alpha h_{b}\left(\frac{I(X;Y)}{2\alpha (M-4)}\right).$$

As before, if $M=e^{R}$, then H(f(X))≤R. Hence,

$$\mathrm{dIB}\left(P_{XY},R\right)\geq I\left(X;Y\right)-2\alpha h_{b}\left(\frac{I(X;Y)}{2\alpha (e^{R}-4)}\right),$$

for all R≥log5. □

## Appendix B. Proofs from Section 3

**Proof** **of Lemma 8.** To prove the upper bound on ${\mathrm{PF}}^{\left(\infty ,1\right)}$, recall that $r\mapsto e^{{\mathrm{PF}}^{\left(\infty ,1\right)}\left(r\right)}$ is convex. Thus, it lies below the chord connecting points (0,0) and $\left(H\left(X\right),e^{I_{\infty }\left(X;Y\right)}\right)$. The lower bound on ${\mathrm{IB}}^{\left(\infty ,1\right)}$ is similarly obtained using the concavity of $R\mapsto e^{{\mathrm{IB}}^{\left(\infty ,1\right)}\left(R\right)}$. This is achievable by an erasure channel. To see this consider the random variable $T_{\delta }$ taking values in X∪{⊥} that is obtained by conditional distributions $P_{T_{\delta }|X}\left(t|x\right)=\bar{\delta }I_{t=x}$ and $P_{T_{\delta }|X}\left(⊥|x\right)=\delta$ for some δ≥0. It can be verified that $I\left(X;T_{\delta }\right)=\bar{\delta }H\left(X\right)$ and $P_{c}\left(Y|T_{\delta }\right)=\bar{\delta }P_{c}\left(Y|X\right)+\delta P_{c}\left(Y\right)$. By taking $\delta =1-\frac{R}{H(X)}$, this channel meets the constraint $I(X;T_{\delta })=R$. Hence,

$${\mathrm{IB}}^{\left(\infty ,1\right)}\left(R\right)\geq \log\left[\frac{P_{c}\left(Y|T_{\delta }\right)}{P_{c}\left(Y\right)}\right]=\log\left[1-\frac{R}{H(X)}+\frac{R}{H(X)}\frac{P_{c}\left(Y|X\right)}{P_{c}\left(Y\right)}\right].$$

□

**Proof** **of Theorem 11.** We begin by ${\mathrm{PF}}^{\left(\infty ,1\right)}$. As described in Section 3.1, and similar to Mrs. Gerber’s Lemma (Lemma 4), we need to construct the lower convex envelope $K_{\cup }\left[F_{\beta }^{\left(\infty ,1\right)}\right]$ of $F_{\beta }^{\left(\infty ,1\right)}\left(q\right)=P_{c}\left(Y^{\prime }\right)+\beta H\left(X^{\prime }\right)$ where $X^{\prime }∼\mathrm{Bernoulli}\left(q\right)$ and $Y^{\prime }$ is the result of passing $X^{\prime }$ through BSC(δ), i.e., $Y^{\prime }∼\mathrm{Bernoulli}\left(\delta *q\right)$. In this case, $P_{c}\left(Y^{\prime }\right)=\max\left\{\delta *q,1-\delta *q\right\}$. Hence, we need to determine the lower convex envelope of the map

(A12) $$q\mapsto F_{\beta }^{\left(\infty ,1\right)}\left(q\right)=\max\left\{\delta *q,1-\delta *q\right\}+\beta h_{b}\left(q\right).$$

A straightforward computation shows that $F_{\beta }^{\left(\infty ,1\right)}\left(q\right)$ is symmetric around $q=\frac{1}{2}$ and is also concave in *q* on $q\in [0,\frac{1}{2}]$ for any β. Hence, $K_{\cup }\left[F_{\beta }^{\left(\infty ,1\right)}\right]$ is obtained as follows depending on the values of β:

- $F_{\beta }^{\left(\infty ,1\right)}\left(\frac{1}{2}\right)<F_{\beta }^{\left(\infty ,1\right)}\left(0\right)$ (see Figure 6a), then $K_{\cup }\left[F_{\beta }^{\left(\infty ,1\right)}\right]$ is given by the convex combination of $F_{\beta }^{\left(\infty ,1\right)}\left(0\right)$, $F_{\beta }^{\left(\infty ,1\right)}\left(1\right)$, and $F_{\beta }^{\left(\infty ,1\right)}\left(\frac{1}{2}\right)$.
- $F_{\beta }^{\left(\infty ,1\right)}\left(\frac{1}{2}\right)=F_{\beta }^{\left(\infty ,1\right)}\left(0\right)$ (see Figure 6b), then $K_{\cup }\left[F_{\beta }^{\left(\infty ,1\right)}\right]$ is given by the convex combination of $F_{\beta }^{\left(\infty ,1\right)}\left(0\right)$, $F_{\beta }^{\left(\infty ,1\right)}\left(\frac{1}{2}\right)$, and $F_{\beta }^{\left(\infty ,1\right)}\left(1\right)$.
- $F_{\beta }^{\left(\infty ,1\right)}\left(\frac{1}{2}\right)>F_{\beta }^{\left(\infty ,1\right)}\left(0\right)$ (see Figure 6c), then $K_{\cup }\left[F_{\beta }^{\left(\infty ,1\right)}\right]$ is given by the convex combination of $F_{\beta }^{\left(\infty ,1\right)}\left(0\right)$ and $F_{\beta }^{\left(\infty ,1\right)}\left(1\right)$.

Hence, assuming $p\leq \frac{1}{2}$, we can construct $T^{*}$ that minimizes $P_{c}\left(Y|T\right)-\beta H\left(X|T\right)$. Considering the first two cases, we obtain that $T^{*}$ is ternary with $P_{X|T^{*}=0}=\mathrm{Bernoulli}\left(0\right)$, $P_{X|T^{*}=1}=\mathrm{Bernoulli}\left(1\right)$, and $P_{X|T^{*}=2}=\mathrm{Bernoulli}\left(\frac{1}{2}\right)$ with marginal $P_{T^{*}}=\left[1-p-\frac{\alpha }{2},p-\frac{\alpha }{2},\alpha \right]$ for some α∈[0,2p]. This leads to $P_{c}\left(Y|T^{*}\right)=\bar{\alpha }\bar{\delta }+\frac{1}{2}\alpha$ and $I\left(X;T^{*}\right)=h_{b}\left(p\right)-\alpha$. Note that $P_{c}\left(Y|T^{*}\right)$ covers all possible domain $\left[P_{c}\left(Y\right),\bar{\delta }\right]$ by varying α on [0,2p]. Replacing $I(X;T^{*})$ by *r*, we obtain $\alpha =h_{b}\left(p\right)-r$ leading to $P_{c}\left(Y|T^{*}\right)=\bar{\delta }-\left(h_{b}\left(p\right)-r\right)\left(\frac{1}{2}-\delta \right)$. Since $P_{c}\left(Y\right)=1-\delta *p$, the desired result follows. To derive the expression for ${\mathrm{IB}}^{\left(\infty ,1\right)}$, recall that we need to derive $K_{\cap }\left[F_{\beta }^{\left(\infty ,1\right)}\right]$ the upper concave envelope of $F_{\beta }^{\left(\infty ,1\right)}$. It is clear from Figure 6 that $K_{\cap }\left[F_{\beta }^{\left(\infty ,1\right)}\right]$ is obtained by replacing $F_{\beta }^{\left(\infty ,1\right)}\left(q\right)$ on the interval $\left[q_{\beta },1-q_{\beta }\right]$ by its maximum value over *q* where

$$q_{\beta }≔\frac{1}{1+e^{\frac{1-2\delta }{\beta }}},$$

is the maximizer of $F_{\beta }^{\left(\infty ,1\right)}\left(q\right)$ on $\left[0,\frac{1}{2}\right]$. In other words,

$$K_{\cap }\left[F_{\beta }^{\left(\infty ,1\right)}\left(q\right)\right]=\left\{\begin{array}{cc} F_{\beta }^{\left(\infty ,1\right)}\left(q_{\beta }\right), & \mathrm{for}\;q\in [q_{\beta },1-q_{\beta }],\\ F_{\beta }^{\left(\infty ,1\right)}\left(q\right), & \mathrm{otherwise}. \end{array}\right.$$

Note that if $p<q_{\beta }$ then $K_{\cap }\left[F_{\beta }^{\left(\infty ,1\right)}\right]$ evaluated at *p* coincides with $F_{\beta }^{\left(\infty ,1\right)}\left(p\right)$. This corresponds to all trivial $P_{T|X}$ such that $P_{c}\left(Y|T\right)+\beta H\left(X|T\right)=P_{c}\left(Y\right)+\beta H\left(X\right)$. If, on the other hand, $p\geq q_{\beta }$, then $K_{\cap }[F_{\beta }^{\left(\infty ,1\right)}$ is the convex combination of $F_{\beta }^{\left(\infty ,1\right)}\left(q_{\beta }\right)$ and $F_{\beta }^{\left(\infty ,1\right)}\left(1-q_{\beta }\right)$. Hence, taking $q_{\beta }$ as a parameter (say, α), the optimal binary $T^{*}$ is constructed as follows: $P_{X|T^{*}=0}=\mathrm{Bernoulli}\left(\alpha \right)$ and $P_{X|T^{*}=1}=\mathrm{Bernoulli}\left(\bar{\alpha }\right)$ for α≤p. Such channel induces

$$P_{c}\left(Y|T^{*}\right)=\max\left\{\alpha *\delta ,1-\alpha *\delta \right\}=1-\alpha *\delta ,$$

as $\alpha \leq p\leq \frac{1}{2}$, and also

$$I\left(X;T^{*}\right)=h_{b}\left(p\right)-h_{b}\left(\alpha \right).$$

Combining these two, we obtain

$$P_{c}\left(Y|T^{*}\right)=1-\delta *h_{b}^{-1}\left(h_{b}\left(p\right)-R\right).$$

□

**Proof** **of Theorem 12.** Let $U_{\alpha }$ and $L_{\alpha }$ denote the $U_{\Phi ,\Psi }$ and $L_{\Phi ,\Psi }$, respectively, when $\Psi \left(Q_{X}\right)=\Phi \left(Q_{X}\right)={∥Q_{X}∥}_{\alpha }$. In light of (59) and (60), it is sufficient to compute $L_{\alpha }$ and $U_{\alpha }$. To do so, we need to construct the lower convex envelope $K_{\cup }\left[F_{\beta }^{\left(\alpha \right)}\right]$ and upper concave envelope $K_{\cap }\left[F_{\beta }^{\left(\alpha \right)}\right]$ of the map $F_{\beta }^{\left(\alpha \right)}\left(q\right)$ given by $q\mapsto ∥Q_{Y}{∥}_{\alpha }-\beta {∥Q_{X}∥}_{\alpha }$ where $X^{\prime }∼\mathrm{Bernoulli}\left(q\right)$ and $Y^{\prime }$ is the result of passing $X^{\prime }$ through BSC(δ), i.e., $Y^{\prime }∼\mathrm{Bernoulli}\left(\delta *q\right)$. In this case, we have

(A13) $$q\mapsto F_{\beta }^{\left(\alpha \right)}\left(q\right)={∥q*\delta ∥}_{\alpha }-{\beta ||q||}_{\alpha },$$

where ${∥a∥}_{\alpha }$ is to mean $∥\left[a,\bar{a}\right]{∥}_{\alpha }$ for any a∈[0,1]. We begin by $L_{\alpha }$ for which we aim at obtaining $K_{\cup }\left[F_{\beta }^{\left(\alpha \right)}\right]$. A straightforward computation shows that $F_{\beta }^{\left(\alpha \right)}\left(q\right)$ is convex for $\beta \leq {\left(1-2\delta \right)}^{2}$ and α≥2. For $\beta >{\left(1-2\delta \right)}^{2}$ and α≥2, it can be shown that $F_{\beta }^{\left(\alpha \right)}\left(q\right)$ is concave an interval $\left[q_{\beta },1-q_{\beta }\right]$ where $q_{\beta }$ solves $\frac{d}{dq}F_{\beta }^{\left(\alpha \right)}\left(q\right)=0$. (The shape of $q\mapsto F_{\beta }^{\left(\alpha \right)}\left(r\right)$ in is similar to what was depicted in Figure 4.) By symmetry, $K_{\cup }\left[F_{\beta }^{\left(\alpha \right)}\right]$ is therefore obtained by replacing $F_{\beta }^{\left(\alpha \right)}\left(q\right)$ on this interval by $F_{\beta }^{\left(\alpha \right)}\left(q_{\beta }\right)$. Hence, if $p<q_{\beta }$, $K_{\cup }\left[F_{\beta }^{\left(\alpha \right)}\right]$ at *p* coincides with $F_{\beta }^{\left(\alpha \right)}\left(p\right)$ which results in trivial $P_{T|X}$ (see the proof of Theorem 11 for more details). If, on the other hand, $p\geq q_{\beta }$, then $K_{\cup }\left[F_{\beta }^{\left(\alpha \right)}\right]$ evaluated at *p* is given by a convex combination of $F_{\beta }^{\left(\alpha \right)}\left(q_{\beta }\right)$ and $F_{\beta }^{\left(\alpha \right)}\left(1-q_{\beta }\right)$. Relabeling $q_{\beta }$ as a parameter (say, *q*), we can write an optimal binary $T^{*}$ via the following: $P_{X|T*=0}=\mathrm{Bernoulli}\left(1-q\right)$ and $P_{X|T*=1}=\mathrm{Bernoulli}\left(q\right)$ for q≤p. This channel induces $\Psi \left(Y|T^{*}\right)={∥q*\delta ∥}_{\alpha }$ and $\Phi \left(X|T^{*}\right)={∥q∥}_{\alpha }$. Hence, the graph of $L_{\alpha }$ is given by

$$\left\{(∥q{∥}_{\alpha },∥q*\delta {∥}_{\alpha }),0\leq q\leq p\right\}.$$

Therefore,

$$\underset{\begin{array}{c} P_{T|X}\\ H_{\alpha }\left(X|T\right)\geq \zeta \end{array}}{\sup}H_{\alpha }\left(Y|T\right)=\frac{\alpha }{1-\alpha }\log{∥q*\delta ∥}_{\alpha },$$

where q≤p solves $\frac{\alpha }{1-\alpha }\log{∥q∥}_{\alpha }=\zeta$. Since the map $q\mapsto {∥q∥}_{\alpha }$ is strictly decreasing for q∈[0,0.5], this equation has a unique solution. Next, we compute $U_{\alpha }$ or equivalently $K_{\cap }\left[F_{\beta }^{\left(\alpha \right)}\right]$ the upper concave envelop of $F_{\beta }^{\left(\alpha \right)}$ defined in (A13). As mentioned earlier, $q\mapsto F_{\beta }^{\left(\alpha \right)}\left(q\right)$ is convex for $\beta \leq {\left(1-2\delta \right)}^{2}$ and α≥2. For $\beta >{\left(1-2\delta \right)}^{2}$, we need to consider three cases: (1) $K_{\cap }\left[F_{\beta }^{\left(\alpha \right)}\right]$ is given by the convex combination of $F_{\beta }^{\left(\alpha \right)}\left(0\right)$ and $F_{\beta }^{\left(\alpha \right)}\left(1\right)$, (2) $K_{\cap }\left[F_{\beta }^{\left(\alpha \right)}\right]$ is given by the convex combination of $F_{\beta }^{\left(\alpha \right)}\left(0\right)$, $F_{\beta }^{\left(\alpha \right)}\left(\frac{1}{2}\right)$, and $F_{\beta }^{\left(\alpha \right)}\left(1\right)$, (3) $K_{\cap }\left[F_{\beta }^{\left(\alpha \right)}\right]$ is given by the convex combination of $F_{\beta }^{\left(\alpha \right)}\left(0\right)$ and $F_{\beta }^{\left(\alpha \right)}\left(q^{†}\right)$ where $q^{†}$ is a point $\in [0,\frac{1}{2}]$. Without loss of generality, we can ignore the first case. The other two cases correspond to the following solutions

- $T^{*}$ is a ternary variable given by $P_{X|T^{*}=0}=\mathrm{Bernoulli}\left(0\right)$, $P_{X|T^{*}=1}=\mathrm{Bernoulli}\left(1\right)$, and $P_{X|T^{*}=2}=\mathrm{Bernoulli}\left(\frac{1}{2}\right)$ with marginal $T^{*}∼\mathrm{Bernoulli}\left(1-p-\frac{\lambda }{2},p-\frac{\lambda }{2},\lambda \right)$ for some λ∈[0,2p]. This produces
  $$\Psi \left(Y|T^{*}\right)=\bar{\lambda }{∥\delta ∥}_{\alpha }+\lambda {∥\frac{1}{2}∥}_{\alpha },$$
  and
  $$\Phi \left(X|T^{*}\right)=\bar{\lambda }+\lambda {∥\frac{1}{2}∥}_{\alpha }.$$
- $T^{*}$ is a binary variable given by $P_{X|T^{*}=0}=\mathrm{Bernoulli}\left(0\right)$ and $P_{X|T^{*}=1}=\mathrm{Bernoulli}\left(\frac{p}{\lambda }\right)$ with marginal $T^{*}∼\mathrm{Bernoulli}\left(\lambda \right)$ for some λ∈[2p,1]. This produces
  $$\Psi \left(Y|T^{*}\right)=\bar{\lambda }{∥\delta ∥}_{\alpha }+\lambda {∥\delta *\frac{p}{\lambda }∥}_{\alpha },$$
  and
  $$\Phi \left(X|T^{*}\right)=\bar{\lambda }+\lambda {∥\frac{p}{\lambda }∥}_{\alpha }.$$

Combining these two cases, can write

$$U_{\alpha }\left(\zeta \right)=\bar{\lambda }{∥\delta ∥}_{\alpha }+\lambda {∥\frac{q}{z}*\delta ∥}_{\alpha },$$

where

$$\zeta =\bar{\lambda }+\lambda {∥\frac{q}{z}∥}_{\alpha },$$

and z=max{2p,λ}. Plugging this into (59) completes the proof. □

**Proof** **of Lemma 9.** The facts that $\gamma \mapsto H_{\gamma }\left(X|T\right)$ is non-increasing on [1,∞] [103] (Proposition 5) and ${\left(\sum_{i}{\left|x_{i}\right|}^{\gamma }\right)}^{1/\gamma }\geq \max_{i}\left|x_{i}\right|$ for all p≥0 imply

(A14) $$\frac{\gamma -1}{\gamma }H_{\gamma }\left(X|T\right)\leq H_{\infty }\left(X|T\right)\leq H_{\gamma }\left(X|T\right).$$

Since $I_{\infty }\left(X;T\right)=H_{\infty }\left(X\right)-H_{\infty }\left(X|T\right)$, the above lower bound yields

(A15) $$I_{\gamma }\left(X;T\right)\geq \frac{\gamma }{\gamma -1}I_{\infty }\left(X;T\right)-\frac{\gamma }{\gamma -1}H_{\infty }\left(X\right)+H_{\gamma }\left(X\right),$$

where the last inequality follows from the fact that $\gamma \mapsto H_{\gamma }\left(X\right)$ is non-increasing. The upper bound in (A14) (after replacing *X* with *Y* and γ with α) implies

(A16) $$I_{\alpha }\left(Y;T\right)\leq I_{\infty }\left(Y;T\right)+H_{\alpha }\left(Y\right)-H_{\infty }\left(Y\right).$$

Combining (A15) and (A16), we obtain the desired upper bound for ${\mathrm{PF}}^{\left(\alpha ,\gamma \right)}$. The other bounds can be proved similarly by interchanging *X* with *Y* and α with γ in (A15) and (A16). □

## References

[1] Tishby N., Pereira F.C., Bialek W. The information bottleneck method. Proceedings of the 37th Annual Allerton Conference on Communication, Control, and Computing.

[2] Wyner A., Ziv J. (1973). A theorem on the entropy of certain binary sequences and applications: Part I. IEEE Trans. Inf. Theory.

[3] Witsenhausen H., Wyner A. (1975). A conditional entropy bound for a pair of discrete random variables. IEEE Trans. Inf. Theory.

[4] Ahlswede R., Körner J. On the connection between the entropies of input and output distributions of discrete memoryless channels. Proceedings of the Fifth Conference on Probability Theory.

[5] Wyner A. (1973). A theorem on the entropy of certain binary sequences and applications—II. IEEE Trans. Inf. Theory.

[6] Kim Y.H., El Gamal A. (2012). Network Information Theory.

[7] Slonim N., Tishby N. Document clustering using word clusters via the information bottleneck method. Proceedings of the 23rd Annual International ACM SIGIR Conference on Research and Development in Information Retrieval.

[8] Still S., Bialek W. (2004). How Many Clusters? An Information-Theoretic Perspective. Neural Comput..

[9] Slonim N., Tishby N. Agglomerative Information Bottleneck. Proceedings of the 12th International Conference on Neural Information Processing Systems (NIPS’99).

[10] Cardinal J. Compression of side information. Proceedings of the 2003 International Conference on Multimedia and Expo—Volume 1.

[11] Zeitler G., Koetter R., Bauch G., Widmer J. Design of network coding functions in multihop relay networks. Proceedings of the 2008 5th International Symposium on Turbo Codes and Related Topics.

[12] Makhdoumi A., Salamatian S., Fawaz N., Médard M. From the Information Bottleneck to the Privacy Funnel. Proceedings of the 2014 IEEE Information Theory Workshop (ITW 2014).

[13] Calmon F.P., Makhdoumi A., Médard M. Fundamental limits of perfect privacy. Proceedings of the IEEE International Symposium on Information Theory (ISIT).

[14] Asoodeh S., Alajaji F., Linder T. Notes on information-theoretic privacy. Proceedings of the 52nd Annual Allerton Conference on Communication, Control, and Computing.

[15] Ding N., Sadeghi P. A Submodularity-based Clustering Algorithm for the Information Bottleneck and Privacy Funnel. Proceedings of the 2019 IEEE Information Theory Workshop (ITW).

[16] Bertran M., Martinez N., Papadaki A., Qiu Q., Rodrigues M., Reeves G., Sapiro G. Adversarially Learned Representations for Information Obfuscation and Inference. Proceedings of the 36th International Conference on Machine Learning.

[17] Lopuhaä-Zwakenberg M., Tong H., Škorić B. (2020). Data Sanitisation Protocols for the Privacy Funnel with Differential Privacy Guarantees. arXiv.

[18] Hsu H., Asoodeh S., Calmon F. Obfuscation via Information Density Estimation. Proceedings of the Twenty Third International Conference on Artificial Intelligence and Statistics.

[19] Dobrushin R., Tsybakov B. (1962). Information transmission with additional noise. IRE Trans. Inf. Theory.

[20] Ahlswede R., Csiszar I. (1986). Hypothesis testing with communication constraints. IEEE Trans. Inf. Theory.

[21] Asoodeh S., Diaz M., Alajaji F., Linder T. (2016). Information extraction under privacy constraints. Information.

[22] Arimoto S., Csiszár I., Elias P. (1977). Information measures and capacity of order *α* for discrete memoryless channels. Topics in Information Theory, Coll. Math. Soc. J. Bolyai.

[23] Raginsky M. (2016). Strong Data Processing Inequalities and Φ-Sobolev Inequalities for Discrete Channels. IEEE Trans. Inf. Theory.

[24] Hsu H., Asoodeh S., Salamatian S., Calmon F.P. Generalizing Bottleneck Problems. Proceedings of the 2018 IEEE International Symposium on Information Theory (ISIT).

[25] Courtade T.A. Strengthening the entropy power inequality. Proceedings of the IEEE International Symposium on Information Theory (ISIT).

[26] Globerson A., Tishby N. (2004). On the Optimality of the Gaussian Information Bottleneck Curve.

[27] Calmon F.P., Polyanskiy Y., Wu Y. Strong data processing inequalities in power-constrained Gaussian channels. Proceedings of the IEEE International Symposium on Information Theory (ISIT).

[28] Rényi A. (1959). On measures of dependence. Acta Math. Acad. Sci. Hung..

[29] Goldfeld Z., Polyanskiy Y. (2020). The Information Bottleneck Problem and its Applications in Machine Learning. IEEE J. Sel. Areas Inf. Theory.

[30] Zaidi A., Estella-Aguerri I., Shamai (Shitz) S. (2020). On the Information Bottleneck Problems: Models, Connections, Applications and Information Theoretic Views. Entropy.

[31] Strouse D., Schwab D.J. (2017). The Deterministic Information Bottleneck. Neural Comput..

[32] Bhatt A., Nazer B., Ordentlich O., Polyanskiy Y. (2018). Information-Distilling Quantizers. arXiv.

[33] Shamir O., Sabato S., Tishby N. (2010). Learning and Generalization with the Information Bottleneck. Theor. Comput. Sci..

[34] Diaz M., Wang H., Calmon F.P., Sankar L. (2020). On the Robustness of Information-Theoretic Privacy Measures and Mechanisms. IEEE Trans. Inf. Theory.

[35] El-Yaniv R., Souroujon O. (2001). Iterative Double Clustering for Unsupervised and Semi-Supervised Learning. Proceedings of the 12th European Conference on Machine Learning.

[36] Elidan G., Friedman N. (2005). Learning Hidden Variable Networks: The Information Bottleneck Approach. J. Mach. Learn. Res..

[37] Aguerri I.E., Zaidi A. (2017). Distributed Information Bottleneck Method for Discrete and Gaussian Sources. arXiv.

[38] Aguerri I.E., Zaidi A. (2019). Distributed Variational Representation Learning. arXiv.

[39] Strouse D., Schwab D.J. (2019). Geometric Clustering with the Information Bottleneck. Neural Comput..

[40] Cicalese F., Gargano L., Vaccaro U. (2018). Bounds on the Entropy of a Function of a Random Variable and Their Applications. IEEE Trans. Inf. Theory.

[41] Koch T., Lapidoth A. (2013). At Low SNR, Asymmetric Quantizers are Better. IEEE Trans. Inf. Theory.

[42] Pedarsani R., Hassani S.H., Tal I., Telatar E. On the construction of polar codes. Proceedings of the 2011 IEEE International Symposium on Information Theory Proceedings.

[43] Tal I., Sharov A., Vardy A. Constructing polar codes for non-binary alphabets and MACs. Proceedings of the 2012 IEEE International Symposium on Information Theory Proceedings.

[44] Kartowsky A., Tal I. (2019). Greedy-Merge Degrading has Optimal Power-Law. IEEE Trans. Inf. Theory.

[45] Viterbi A.J., Omura J.K. (1979). Principles of Digital Communication and Coding.

[46] Tishby N., Zaslavsky N. Deep learning and the information bottleneck principle. Proceedings of the IEEE Information Theory Workshop (ITW).

[47] Shwartz-Ziv R., Tishby N. (2017). Opening the Black Box of Deep Neural Networks via Information. arXiv.

[48] Saxe A.M., Bansal Y., Dapello J., Advani M., Kolchinsky A., Tracey B.D., Cox D.D. On the Information Bottleneck Theory of Deep Learning. Proceedings of the International Conference on Learning Representations.

[49] Goldfeld Z., Van Den Berg E., Greenewald K., Melnyk I., Nguyen N., Kingsbury B., Polyanskiy Y. Estimating Information Flow in Deep Neural Networks. Proceedings of the 36th International Conference on Machine Learning.

[50] Amjad R.A., Geiger B.C. (2020). Learning Representations for Neural Network-Based Classification Using the Information Bottleneck Principle. IEEE Trans. Pattern Anal. Mach. Intell..

[51] Alemi A.A., Fischer I., Dillon J.V., Murphy K. (2016). Deep Variational Information Bottleneck. arXiv.

[52] Kolchinsky A., Tracey B.D., Wolpert D.H. (2017). Nonlinear Information Bottleneck. arXiv.

[53] Kolchinsky A., Tracey B.D., Kuyk S.V. Caveats for information bottleneck in deterministic scenarios. Proceedings of the International Conference on Learning Representations.

[54] Chalk M., Marre O., Tkacik G. (2016). Relevant Sparse Codes with Variational Information Bottleneck. Proceedings of the 30th International Conference on Neural Information Processing Systems (NIPS’16).

[55] Wickstrøm K., Løkse S., Kampffmeyer M., Yu S., Principe J., Jenssen R. (2019). Information Plane Analysis of Deep Neural Networks via Matrix–Based Rényi’s Entropy and Tensor Kernels. arXiv.

[56] Matias V., Piantanida P., Rey Vega L. The Role of the Information Bottleneck in Representation Learning. Proceedings of the IEEE International Symposium on Information Theory (ISIT 2018).

[57] Alemi A., Fischer I., Dillon J. (2018). Uncertainty in the Variational Information Bottleneck. arXiv.

[58] Yu S., Jenssen R., Príncipe J. (2018). Understanding Convolutional Neural Network Training with Information Theory. arXiv.

[59] Cheng H., Lian D., Gao S., Geng Y. Evaluating Capability of Deep Neural Networks for Image Classification via Information Plane. Proceedings of the ECCV.

[60] Higgins I., Matthey L., Pal A., Burgess C., Glorot X., Botvinick M., Mohamed S., Lerchner A. *β*-VAE: Learning Basic Visual Concepts with a Constrained Variational Framework. Proceedings of the ICLR.

[61] Issa I., Wagner A.B., Kamath S. (2020). An Operational Approach to Information Leakage. IEEE Trans. Inf. Theory.

[62] Cvitkovic M., Koliander G. Minimal Achievable Sufficient Statistic Learning. Proceedings of the 36th International Conference on Machine Learning.

[63] Asoodeh S., Alajaji F., Linder T. On maximal correlation, mutual information and data privacy. Proceedings of the IEEE 14th Canadian Workshop on Inf. Theory (CWIT).

[64] Makhdoumi A., Fawaz N. Privacy-utility tradeoff under statistical uncertainty. Proceedings of the 51st Allerton Conference on Communication, Control, and Computing.

[65] Asoodeh S., Diaz M., Alajaji F., Linder T. (2019). Estimation Efficiency Under Privacy Constraints. IEEE Trans. Inf. Theory.

[66] Asoodeh S., Diaz M., Alajaji F., Linder T. Privacy-aware guessing efficiency. Proceedings of the IEEE International Symposium on Information Theory (ISIT).

[67] Asoodeh S., Alajaji F., Linder T. Privacy-aware MMSE estimation. Proceedings of the IEEE International Symposium on Information Theory (ISIT).

[68] Calmon F.P., Makhdoumi A., Médard M., Varia M., Christiansen M., Duffy K.R. (2017). Principal Inertia Components and Applications. IEEE Trans. Inf. Theory.

[69] Wang H., Vo L., Calmon F.P., Médard M., Duffy K.R., Varia M. (2019). Privacy With Estimation Guarantees. IEEE Trans. Inf. Theory.

[70] Asoodeh S. (2017). Information and Estimation Theoretic Approaches to Data Privacy. Ph.D. Thesis.

[71] Liao J., Kosut O., Sankar L., du Pin Calmon F. (2019). Tunable Measures for Information Leakage and Applications to Privacy-Utility Tradeoffs. IEEE Trans. Inf. Theory.

[72] Duchi J.C., Jordan M.I., Wainwright M.J. (2014). Privacy aware learning. J. Assoc. Comput. Mach. (ACM).

[73] Poole B., Ozair S., Van Den Oord A., Alemi A., Tucker G. On Variational Bounds of Mutual Information. Proceedings of the 36th International Conference on Machine Learning.

[74] Belghazi M.I., Baratin A., Rajeshwar S., Ozair S., Bengio Y., Courville A., Hjelm D. Mutual Information Neural Estimation. Proceedings of the 35th International Conference on Machine Learning.

[75] Van den Oord A., Li Y., Vinyals O. (2018). Representation Learning with Contrastive Predictive Coding. arXiv.

[76] Song J., Ermon S. Understanding the Limitations of Variational Mutual Information Estimators. Proceedings of the International Conference on Learning Representations.

[77] McAllester D., Stratos K. Formal Limitations on the Measurement of Mutual Information. Proceedings of the International Conference on Learning Representations.

[78] Rassouli B., Gunduz D. On Perfect Privacy. Proceedings of the 2018 IEEE International Symposium on Information Theory (ISIT).

[79] Csiszár I., Körner J. (2011). Information Theory: Coding Theorems for Discrete Memoryless Systems.

[80] Kim H., Gao W., Kannan S., Oh S., Viswanath P., Guyon I., Luxburg U.V., Bengio S., Wallach H., Fergus R., Vishwanathan S., Garnett R. (2017). Discovering Potential Correlations via Hypercontractivity. Advances in Neural Information Processing Systems 30.

[81] Ahlswede R., Gács P. (1976). Spreading of sets in product spaces and hypercontraction of the Markov operator. Ann. Probab..

[82] Anantharam V., Gohari A., Kamath S., Nair C. (2014). On maximal correlation, hypercontractivity, and the data processing inequality studied by Erkip and Cover. arXiv.

[83] Polyanskiy Y., Wu Y. (2016). Dissipation of Information in Channels With Input Constraints. IEEE Trans. Inf. Theory.

[84] Chechik G., Globerson A., Tishby N., Weiss Y. (2005). Information Bottleneck for Gaussian Variables. J. Mach. Learn. Res..

[85] Zaidi A. Hypothesis Testing Against Independence Under Gaussian Noise. Proceedings of the 2020 IEEE International Symposium on Information Theory (ISIT).

[86] Wu T., Fischer I., Chuang I.L., Tegmark M. (2019). Learnability for the Information Bottleneck. Entropy.

[87] Contento L., Ern A., Vermiglio R. (2015). A linear-time approximate convex envelope algorithm using the double Legendre-Fenchel transform with application to phase separation. Comput. Optim. Appl..

[88] Lucet Y. (1997). Faster than the Fast Legendre Transform, the Linear-time Legendre Transform. Numer. Algorithms.

[89] Witsenhausen H. (1980). Indirect rate distortion problems. IEEE Trans. Inf. Theory.

[90] Wyner A. (1975). On source coding with side information at the decoder. IEEE Trans. Inf. Theory.

[91] Courtade T.A., Weissman T. (2014). Multiterminal Source Coding Under Logarithmic Loss. IEEE Trans. Inf. Theory.

[92] Li C.T., El Gamal A. (2018). Extended Gray-Wyner System With Complementary Causal Side Information. IEEE Trans. Inf. Theory.

[93] Vera M., Rey Vega L., Piantanida P. (2019). Collaborative Information Bottleneck. IEEE Trans. Inf. Theory.

[94] Gilad-Bachrach R., Navot A., Tishby N. (2003). An Information Theoretic Tradeoff between Complexity and Accuracy. Learning Theory and Kernel Machines.

[95] Pichler G., Koliander G. Information Bottleneck on General Alphabets. Proceedings of the 2018 IEEE International Symposium on Information Theory (ISIT).

[96] Kim Y.H., Sutivong A., Cover T. (2008). State mplification. IEEE Trans. Inf. Theory.

[97] Merhav N., Shamai S. (2007). Information rates subject to state masking. IEEE Trans. Inf. Theory.

[98] Witsenhausen H. (1980). Some aspects of convexity useful in information theory. IEEE Trans. Inf. Theory.

[99] Harremoës P., Tishby N. The information bottleneck revisited or how to choose a good distortion measure. Proceedings of the IEEE International Symposium on Information Theory (ISIT).

[100] Hirche C., Winter A. An alphabet size bound for the information bottleneck function. Proceedings of the IEEE International Symposium on Information Theory (ISIT).

[101] Liese F., Vajda I. (2006). On Divergences and Informations in Statistics and Information Theory. IEEE Trans. Inf. Theory.

[102] Verdú S. *α*-mutual information. Proceedings of the Information Theory and Applications Workshop (ITA).

[103] Fehr S., Berens S. (2014). On the Conditional Rényi Entropy. IEEE Trans. Inf. Theory.

[104] Csiszár I. (1967). Information-type measures of difference of probability distributions and indirect observation. Stud. Sci. Math. Hung..

[105] Sason I., Verdú S. (2016). *f*-Divergence Inequalities. IEEE Trans. Inf. Theory.

[106] Guntuboyina A., Saha S., Schiebinger G. (2014). Sharp Inequalities for *f*-Divergences. IEEE Trans. Inf. Theory.

[107] Guo D., Shamai S., Verdú S. (2005). Mutual information and minimum mean-square error in Gaussian channels. IEEE Trans. Inf. Theory.

[108] Rockafellar R.T. (1997). Convex Analysis.

[109] Cover T.M., Thomas J.A. (2006). Elements of Information Theory.

[110] Linder T., Zamir R. (2008). On the asymptotic tightness of the Shannon lower bound. IEEE Trans. Inf. Theory.

[111] Guo D., Wu Y., Shitz S.S., Verdú S. (2011). Estimation in Gaussian Noise: Properties of the Minimum Mean-Square Error. IEEE Trans. Inf. Theory.

[112] Jana S. Alphabet sizes of auxiliary random variables in canonical inner bounds. Proceedings of the 43rd Annual Conference on Information Sciences and Systems.

